# Enhanced gating efficiency in vertical mixed molecular transistors with deep orbital level

**DOI:** 10.1126/sciadv.adt3603

**Published:** 2025-06-18

**Authors:** Donguk Kim, Hyemin Lee, Minwoo Song, Jongwoo Nam, Changjun Lee, Jaeyong Woo, Juntae Jang, Minsu Jeong, Hyeonwoo Yeo, Ryong-Gyu Lee, Eunje Park, Hyeonmin Choi, Yong-Hoon Kim, Keehoon Kang, Takhee Lee

**Affiliations:** ^1^ Department of Physics and Astronomy, and Institute of Applied Physics, Seoul National University, Seoul 08826, Korea.; ^2^ School of Electrical Engineering, Korea Advanced Institute of Science and Technology (KAIST), Daejeon 34141, Korea.; ^3^ Department of Materials Science and Engineering, Research Institute of Advanced Materials, Soft Foundry Institute and Institute of Applied Physics, Seoul National University, Seoul 08826, Korea.

## Abstract

The advancement of molecular junction transistors relies heavily on precise modulation of molecular orbitals, yet this is hindered by a limited transmission window and reduced bias stability, which typically restricts the range of active channel molecules adopted to those with orbital levels near Fermi level of the contacts. In this study, we demonstrate an effective orbital gating of prototypical alkanethiol–based molecules with deeper orbital levels in vertical large-area mixed self-assembled monolayers (SAMs) configuration that offers enhanced electrical bias stability and gating efficiency. By using ion gel gating in Au-molecule-graphene junction, the channel conductance could be modulated notably according to a clear transition from direct tunneling to Fowler-Nordheim tunneling regime. The mixed SAM molecular transistors also showed a superior gating efficiency due to the suppressed field screening effect by the net molecular dipole. This work is expected to contribute toward developing reliable three-terminal molecular device platform extended to molecules with deep orbital levels.

## Teaser

Improved gating efficiency via mixed SAM unlocks the hidden potential of molecular electronics.

## INTRODUCTION

Efforts toward the ultimate miniaturization of electronic devices have intensified along with molecular electronics research (*1*–*5*). These efforts have explored the use of molecules as electronic components, including diodes (*6*–*8*), thermoelectric devices (*9*–*11*), transistors (*12*–*18*), and neuromorphic devices (*19*–*21*), with single-molecule and large-area ensemble molecular junctions being used as the two main testbeds (*4*). In particular, the development of molecular junction transistor, i.e., a three-terminal device that is able to modulate the channel conductance along the molecule via an effective modulation of molecular orbitals (i.e., orbital gating), remains a prominent challenge in the field of molecular electronics. Single-molecule transistor devices can use a simple planar structure with conventional electrostatic gating through a gate dielectric (*12*, *13*, *22*, *23*), as well as electrochemical gating (*14*, *15*, *24*–*26*). In large-area molecular transistor devices, ionic electrostatic gating and a gate-field permeable top electrode, such as graphene, are commonly used in a vertical device structure (*16*–*18*).

Although orbital gating is expected to be a general phenomenon in molecular transistors, previous studies have mainly focused on a specific range of molecules with a relatively narrow highest occupied molecular orbital (HOMO)–lowest unoccupied molecular orbital (LUMO) gap (such as aromatic molecules), positioning the frontier orbitals close to the Fermi level of the contact electrodes. A summary of gate efficiencies, along with their corresponding device structures and gating methods, is provided in table S1 (*12*–*18*, *24*–*26*). This reflects the restricted voltage bias range that can be applied before electrical breakdown occurs and also the limited tunable transmission window for charge transport, as governed by the established Landauer formalism (*27*, *28*).

To address this issue, we have used mixed molecules self-assembled monolayer (SAM) junction configuration that consists of matrix and reinforced molecules for developing molecular transistors that can accommodate a wider voltage bias window for orbital gating (*29*, *30*). The alkanethiol-based mixed molecules with deep frontier orbital levels were used; 16-mercaptohexadecenoic acid (16MHDA) as the matrix molecule and shorter dodecanethiol (C12) molecules as the reinforcement molecule, both of which self-assembled on bottom Au contact to form electrically stable junctions. Building on this approach, our molecular junction transistor devices in a vertical Au-molecule-graphene structure with ion gel gating demonstrates an effective orbital gating as shown from the tunability of the channel conductance according to different transport regimes, as confirmed by transition voltage spectroscopy (TVS). In addition, the asymmetric conductance of the mixed SAM devices with respect to the drain bias polarity as represented by a significant rectification ratio can be modulated by the applied gate voltage. Furthermore, a suppressed field screening in graphene caused by the net molecular dipole led to an enhanced gating efficiency in mixed SAM devices. This work could facilitate the development of a general device platform for molecular junction transistors that can operate on the basis of an effective orbital gating of molecular channels not only limited to aromatic molecules but those with deep orbital levels capable of accommodating transition between distinct molecular transport regimes.

## RESULTS

### Designing three-terminal mixed SAM molecular junction

**Fig. 1.**
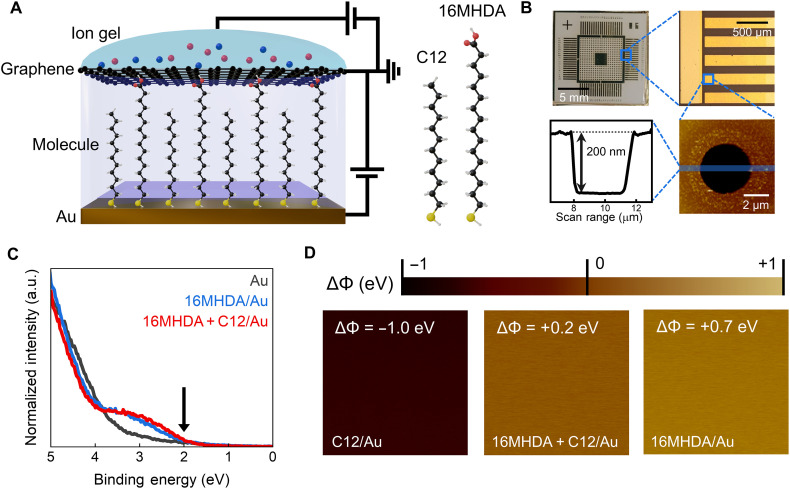
Schematic of molecular transistor and characterization of molecules. (**A**) A schematic of the molecular junction is shown with chemical structures of molecules. The longer molecule, 16MHDA, acts as matrix molecule, while shorter one, C12, acts as reinforcement molecule. (**B**) A series of optical images (top) and AFM image (bottom right) of molecular transistor devices. Cross-sectional depth profile (bottom left) along the line across the hole in AFM image is shown. (**C**) UPS data of Au, single-type SAM (16MHDA/Au) and mixed SAM (16MHDA + C12/Au) samples. Onset of HOMO level of around 2 eV (indicated with an arrow) was observed for both single-type and mixed SAMs. a.u., arbitrary units. (**D**) KPFM data showing the effect of molecular dipoles on the work function of Au substrates with SAMs of C12, 16MHDA, and 16MHDA + C12 over the scanning area of 1 μm^2^. Color bar shows relative disparity (ΔΦ) in work function compared to that of Au (5.1 eV).

To facilitate gate modulation of molecular transistors, we used a vertical molecular device structure made with SAMs, as shown in Fig. 1 (A and B). In these devices, Au serves as the bottom electrode to which the drain voltage is applied, while monolayer graphene functions as the top electrode with grounded source voltage. Gate modulation is achieved by applying an electrostatic potential to the ion gel on top of the graphene top electrode. Photoresist (PR) is used as an insulating wall between the electrodes to form a via hole that defines the molecular active channel. On the Au bottom electrode within the holes, molecules are self-assembled in a homogenous manner (single-type molecule) or heterogenous manner (mixed molecules). Heterogenous molecular junctions are created by so-called repeated surface exchange of molecules (ReSEM) method, which can form mixed SAM molecular junctions without phase segregation (*31*, *32*). It has also been reported that the mixed SAM molecular junctions made with the ReSEM method produces molecular junctions capable of enduring high voltage biases (up to several volts) (*29*, *30*). The fabrication process of these molecular devices is explained in detail in fig. S1. Note that several researchers have also demonstrated SAM field effect transistors (SAM FETs) (*33*–*35*), which can be distinguished from the proposed vertical molecular junction architecture of this study. SAM FETs are three-terminal devices where the conduction occurs via lateral hopping transport between overlapping functional groups of SAM molecules rather than along the molecular chains.

Figure 1A illustrates a cross-sectional schematic of the molecular junction, indicating that the junction contains longer 16-mercaptohexadecenoic acid (denoted as 16MHDA) molecules and shorter dodecanethiol (denoted as C12) molecules. These alkane-based molecules typically have deeper orbital levels compared to aromatic-based molecules (*36*). The chemical structures of 16MHDA and C12 are also shown in Fig. 1A. Figure 1B displays images of the molecular device under increasing magnification, where the radius and depth of the hole in the PR are determined to be ~2.5 μm and ~200 nm, respectively, with atomic force microscopy (AFM). Figure 1C shows the ultraviolet photoelectron spectroscopy (UPS) data measured on bare Au, 16MHDA on Au, and mixed 16MHDA and C12 (denoted as 16MHDA + C12) on Au. The onset of the HOMO level for both 16MHDA SAM and 16MHDA + C12 mixed SAM was found to be similar, which is evidenced by an increase in intensity at ~2 eV (Fermi level is located at 0 eV).

The effect of dipole modulation to work functions by molecular SAMs can be investigated through Kelvin probe force microscopy (KPFM) measurements and presented in Fig. 1D. Figure 1D presents the relative disparity (ΔΦ) in work function compared to that of the Au tip. This difference is attributed to the molecular dipoles that modify the work function of the Au substrate. C12 SAM on Au substrate produces dipole that lowers the energy of the vacuum level (by −1.0 eV), whereas 16MHDA SAM results in an upward shift of the vacuum level (by 0.7 eV) (fig. S4). In addition, performing density functional theory (DFT) calculations, we obtained the work function modulation values of −0.49 and +0.87 eV for C12 and 16MHDA cases, respectively, which are in good agreement with the KPFM measurement data (see section S3 and figs. S7 to S9). The change of the vacuum level by the dipole of the mixed SAM lies intermediate to those of each SAM (individual 16MHDA and C12 SAMs), which confirms the coexistence of both molecular types in the mixed SAM configuration (*37*).

### Electrical measurement of two-terminal molecular junctions

**Fig. 2.**
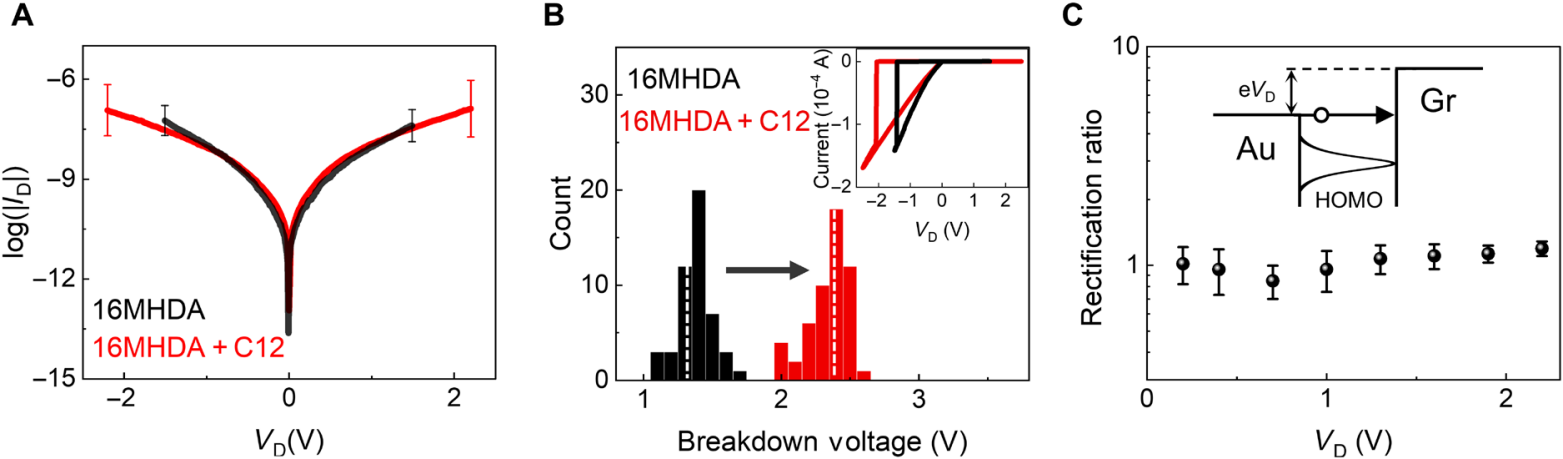
Electrical characterization of molecular junctions. (**A**) Logarithmic plot of current-voltage data for 16MHDA SAM and 16MHDA + C12 SAM junctions. Average data and error bars were obtained from statistical measurement of about 30 different molecular junctions for each type. (**B**) Histogram of breakdown voltages for 16MHDA and 16MHDA + C12 devices. White dashed lines show mean breakdown voltage values for each configuration. Inset shows typical current-voltage breakdown characteristics. (**C**) Rectification ratio [= *I*(+V)/*I*(−V)] at different drain voltages. Inset graphic shows direct tunneling transport scheme. Gr represents graphene electrode.

We conducted a comparative analysis of the current-voltage characteristics for single-type SAM and mixed SAM molecular junctions. Figure 2A shows the current-voltage (*I*-*V*_D_) data for 16MHDA SAM and 16MHDA + C12 mixed SAM molecular junctions. For a statistical analysis, more than 30 molecular devices for each type were characterized and the average *I*-*V*_D_ curves are shown in Fig. 2A with error bars. The *I*-*V*_D_ data for C12 SAM molecular devices are shown in fig. S10A. The electrical characteristics of the 16MHDA and 16MHDA + C12 devices are similar up to 1.5 V. For the 16MHDA + C12 devices, the applied voltage was extended to 2.2 V; however, the applied voltage was limited to 1.5 V for the 16MHDA devices because of breakdown occurring at voltages exceeding 1.5 V. A typical breakdown behavior in *I*-*V*_D_ data is presented in the inset of Fig. 2B and fig. S10B, exhibiting a sudden surge in current to several hundred microampere. Figure 2B shows the histogram of the breakdown voltages for 16MHDA and 16MHDA + C12 devices. The average breakdown voltages were found to be ~1.3 and ~2.4 V for 16MHDA and 16MHDA + C12 devices, respectively. The breakdown voltage for the mixed SAM junctions exceeds that of single-type SAM junctions by ~1 V, which is in accordance with previous studies that showed that the mixed SAMs produce better ordered junctions that are stable up to higher voltages (*29*, *30*).

We investigated symmetricity of current under the positive and negative voltages. The rectification ratios, defined the ratio of current [= *I*(+|*V*_D_|)/*I*(−|*V*_D_|)], are shown in Fig. 2C. The 16MHDA + C12 devices exhibited a nonrectifying response, maintaining a rectification ratio of unity for the applied voltage up to 2.2 V. This observation aligns with previous studies that indicate that the HOMO and LUMO levels are distanced substantially away from the Fermi level, corroborating the UPS data in Fig. 1C. The insufficient voltage fails to shift the molecular orbital levels into the transmission window (inset of Fig. 2C), resulting in a direct tunneling transport mechanism regardless of the polarity of the applied voltage and thus nonrectifying response. The confirmation of this transport mechanism is further supported by the absence of a correlation between 1/T (1/K) and log(J) in the Arrhenius plot (fig. S12), indicating that direct tunneling is the dominant transport mechanism. This conclusion arises from the governing equation for direct tunneling, which does not explicitly include temperature-dependent terms.

### Gate-induced modulation of molecular orbitals

**Fig. 3.**
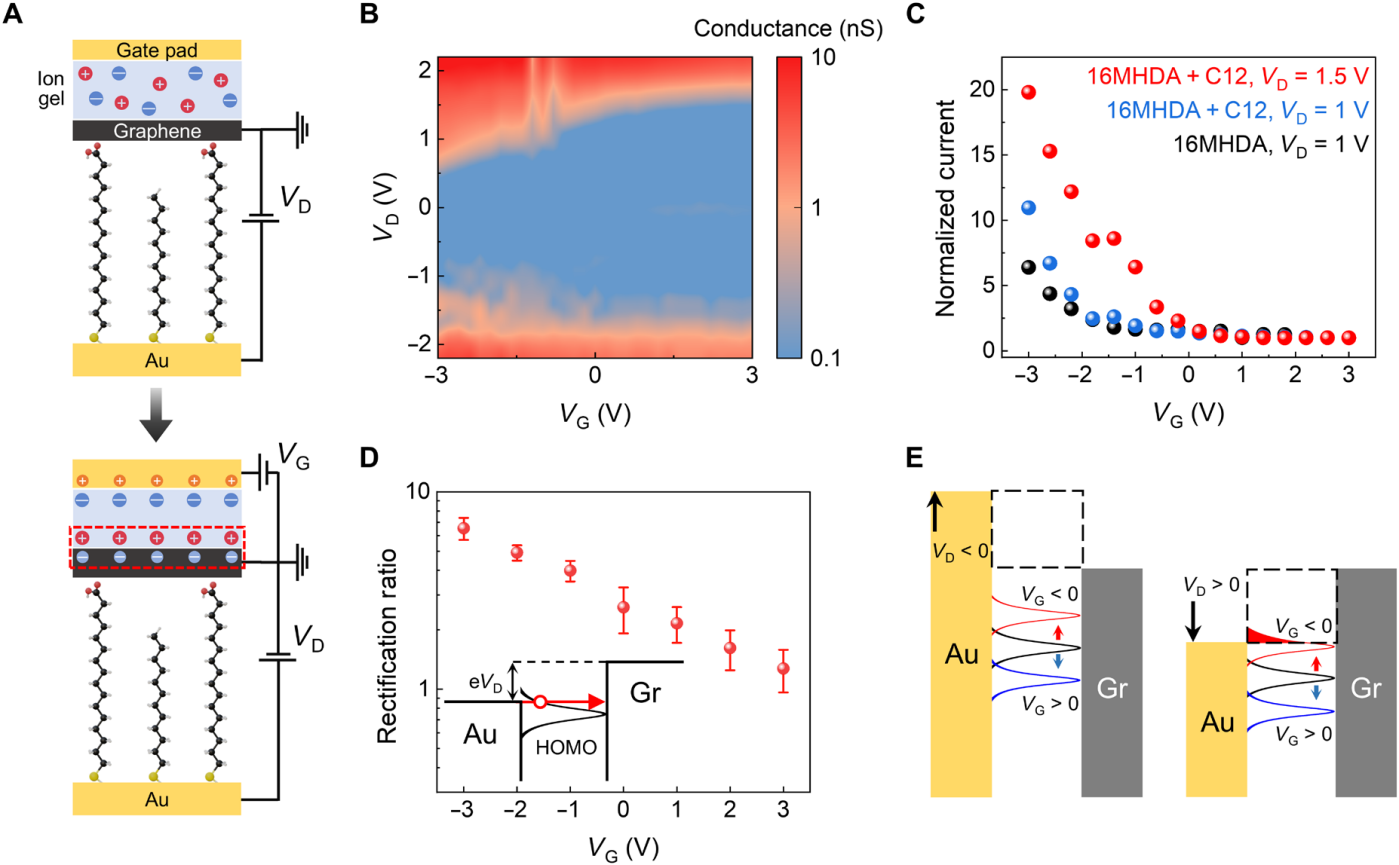
Gate modulation of molecular transistors. (**A**) Illustration of molecular transistor junctions with using ion gel without (top) and with gate voltage (bottom). Electrical double layer (marked as a red dashed box) is formed atop the graphene electrode with an applied gate voltage. Cations and anions are represented by red and blue circles, respectively. (**B**) Contour plot of conductance (d*I*/d*V*_D_) as functions of *V*_D_ and *V*_G_ for a 16MHDA + C12 transistor. (**C**) Normalized current versus gate voltage for 16MHDA and 16MHDA + C12 at *V*_D_ = 1 V, along with data for 16MHDA + C12 at *V*_D_ = 1.5 V. (**D**) Rectification ratio [*I*(+|*V*_D_ = 2.2 V|)/*I*(−|*V*_D_ = 2.2 V|)] values plotted for different gate voltages. Error bars indicate SDs. (**E**) Schematics of molecular junctions. Molecular orbitals do not enter and enter the transmission window under negative and positive drain voltages, respectively. Dashed boxes indicate transmission window.

We fabricated three-terminal molecular transistors by using an ion gel (1-ethyl-3-methylimidazolium bis(trifluoromethylsulfonyl)imide; EMIM-TFSI) onto the molecular junctions of mixed SAMs. Figure 3A shows the schematics of molecular junctions with and without applied gate voltage (V_G_). The bottom schematic of Fig. 3A demonstrates the creation of an electrical double layer atop the monolayer graphene electrode, induced by applying a gate voltage to the gate pad of the molecular junctions, which facilitates molecular orbital gating in this study. Owing to the partial electrical transparency of graphene to electric field (*38*), this electrical double layer, composed of cations and anions, generates localized electric field that penetrates into the molecular orbitals, thus adjusting their energy levels. Figure 3B shows a contour plot of conductance (d*I*/d*V*_D_) as functions of *V*_D_ and *V*_G_. The conductance increases monotonically as the gate voltage is swept from +3 to −3 V, exhibiting p-type behavior indicative of HOMO-dominated transport, consistent with the characteristics of single-type SAM devices (fig. S15). In particular, there is a more pronounced increase in current at positive drain voltages compared to negative drain voltages (fig. S16). Figure 3C presents the transfer characteristics of single-type and mixed SAM transistors, with current levels normalized by dividing the currents by the value at *V*_G_ = +3 V. At a drain voltage of 1 V, mixed SAM transistors exhibit an on/off ratio of approximately 11, higher than the ~6 observed in single-type SAM transistors. In addition, the broader drain voltage window in mixed SAM transistors, as shown in Fig. 2A, allowed for the analysis of transfer curves at *V*_D_ = 1.5 V, revealing even larger on/off ratios, with the ratio reaching approximately 20, demonstrating effective gate modulation for the deep orbital level molecule 16MHDA.

Figure 3D shows the rectification ratios [*I*(+|*V*_D_ = 2.2 V|)/*I*(−|*V*_D_ = 2.2 V|)] as a function of gate voltage. The rectification ratio at *V*_G_ = 0 V does not achieve unity unlike the result observed in Fig. 2C (also see fig. S14). Furthermore, the rectification ratio depends on the gate voltage, showing larger value as more negative gate voltage is applied. This phenomenon can be explained by the energy band alignment with respect to the Fermi levels of the source and drain electrodes, as shown in Fig. 3E. Under negative drain voltages, the molecular orbital levels remain outside the transmission window, which results in a direct tunneling as a primary transport mechanism throughout gate voltages applied in this study (left side of Fig. 3E). Conversely, the application of a positive drain voltage causes an upward shift in the molecular orbital level that couples strongly with the graphene electrode, as the localized HOMO of 16MHDA moiety is in van der Waals contact with graphene but is separated from the Au electrode by the alkane chain of 16MHDA (*39*). In this case, a negative gate voltage elevates the HOMO level of the 16MHDA molecule, allowing it to cross into the transmission window (marked as a black dashed box) in conjunction with the positive drain voltage (right side of Fig. 3E) and leading to a more asymmetric current response. In section S7, on the basis of multi-space constrained-search DFT (MS-DFT) calculation (*40*–*43*) results, we provide the mechanistic explanation of the gating mechanisms including the role of graphene electrode.

### TVS analysis and gating efficiencies of molecular transistors

**Fig. 4.**
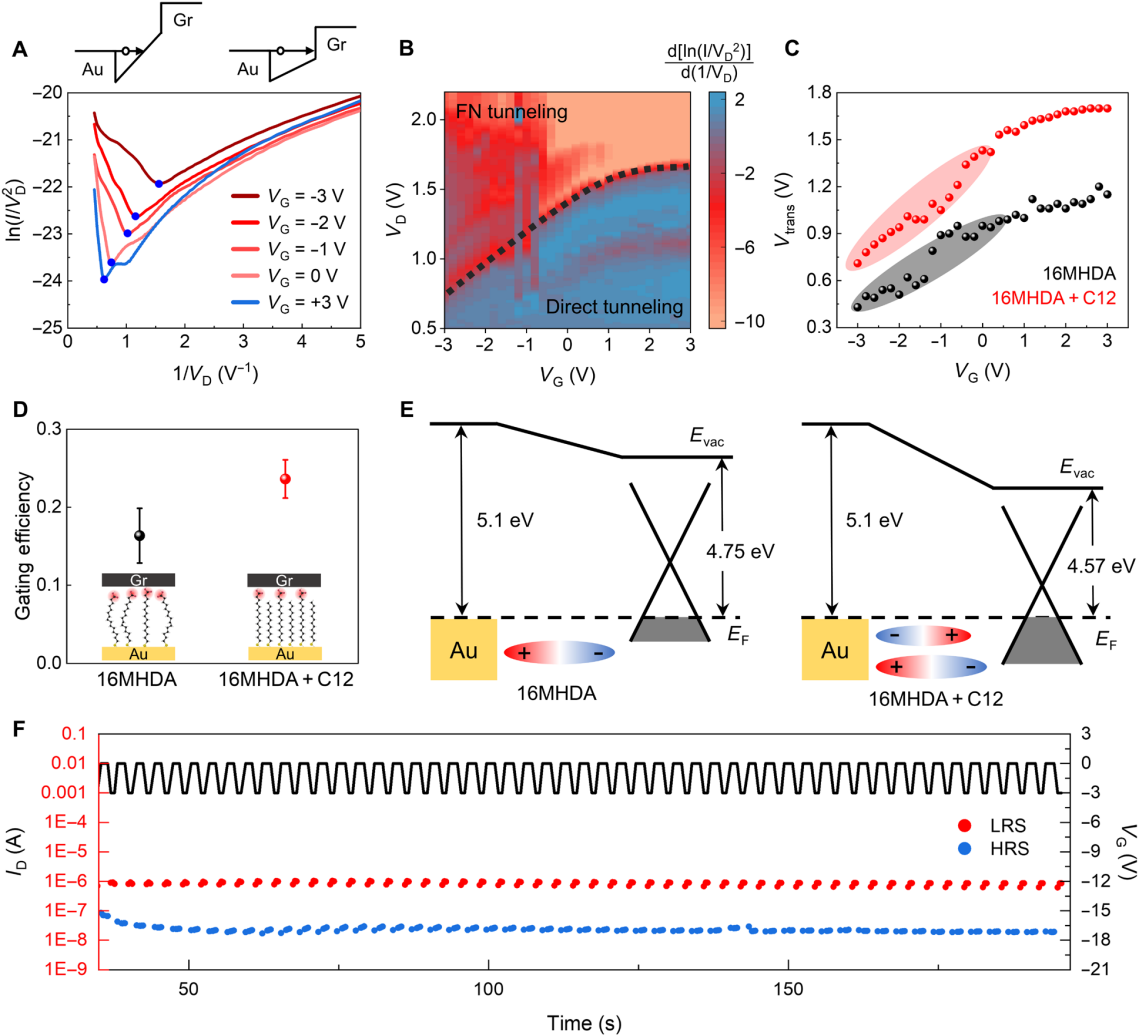
TVS analysis in molecular transistors. (**A**) TVS analysis for a 16MHDA + C12 molecular transistor. (**B**) Heatmap of slope values of TVS curves as functions of *V*_D_ and *V*_G_. The region (blue color) of positive slope values below the black dotted line indicates that direct tunneling dominates, and the negative slope region (red color) above the dotted line indicates that F-N tunneling dominates. (**C**) Transition voltage acquired from TVS analysis at different gate voltages for 16MHDA and 16MHDA + C12 molecular transistors. The slope in this plot represents the gating efficiency, with the colored regions highlighting the negative gate voltage areas. (**D**) Average gating efficiency values for 16MHDA and 16MHDA + C12 molecular transistors. (**E**) Molecular dipole and its effect on the Fermi level of graphene are presented for single-type 16MHDA SAM and mixed 16MHDA + C12 SAM junctions. Mixed SAM junction incorporates smaller dipole resulting in less modulation in the Fermi level of graphene. (**F**) Current response of a 16MHDA + C12 molecular transistor when gate voltage pulses alternating between 0 and −3 V are applied at drain voltage of +2 V.

Figure 4A shows the graph of ln(*I*/$V_{D}^{2}$ ) versus 1/*V*_D_, known as a Fowler-Nordheim (F-N) plot. Charge transport is dominated by direct tunneling at low *V*_D_ and F-N tunneling at high *V*_D_ regime, as illustrated in the schematics in Fig. 4A (top). Direct tunneling refers to charge transport through a rectangular barrier at low electric fields, whereas F-N tunneling involves field-assisted charge transport through a triangular barrier at high electric fields. As *V*_D_ increases, charge transport changes from direct to F-N tunneling accompanied by a change in slope from positive to negative with the stationary points called the transition voltages (*V*_Trans_) marked by blue dots in Fig. 4A. As more negative gate voltage is applied, *V*_Trans_ shifts to a lower voltage, signifying the decrease in tunneling barrier height. This confirms that the charge transport occurs mainly through the HOMO of the molecule rather than its LUMO. Figure 4B presents a heatmap of the slope values of TVS curves as a function of drain and gate voltages. The heatmap of Fig. 4B distinctly shows the *V*_Trans_ line (black dotted line) at which the TVS curve’s slope changes with increasing drain voltage, indicating the onset of F-N tunneling. The region of positive slope values below the *V*_Trans_ line means that direct tunneling is the primary charge transport mechanism. On the other hand, the negative slope region above the *V*_Trans_ line means that F-N tunneling predominates as the transport mechanism. This demonstrates that gate modulation of molecular orbitals at a fixed drain voltage can switch the transport mechanism between direct and F-N tunneling, which underlies the transistor on/off behavior observed in Fig. 3. The gating efficiency can be defined as Δ*V*_Trans_/Δ*V*_G_, which describes the effectiveness of molecular orbital gating. The gating efficiency was estimated to be 0.24 at negative gate voltage range (−3 to 0 V), but it was found to be lower at positive gate voltage range (Fig. 4B). The weaker gating efficiency at positive gate voltages can be associated with the variations in the electrical double layer length due to different molecular sizes of cations and anions (cation being larger than the anion) (*44*). This leads to increased density of anions compared to cations at surface charge layer and results in stronger electric field subjected to molecular orbitals at negative gate voltages (see fig. S13).

The gating efficiencies (slopes of curves in Fig. 4C) were also compared between single-type 16MHDA SAM and mixed 16MHDA + C12 SAM devices. Mixed SAM devices showed steeper slope, i.e., higher gating efficiency than single-type SAM devices. The gating efficiency values (obtained from negative gate voltage range of −3 to 0 V) are presented in Fig. 4D, in which the average values and error bars were obtained from measurement of three different devices for each case. The gating efficiency values were found to be 0.16 and 0.24 for single-type SAM and mixed SAM devices, respectively. This improvement of gating efficiency is related to the molecular dipoles presented in Fig. 1D and fig. S4. For 16MHDA + C12/Au, the negative (toward Au) dipole of 16MHDA is counterbalanced by the strong positive (away from Au) dipole of C12, resulting in an overall weak negative dipole. In single-type 16MHDA SAM, the large negative dipole induces stronger electrostatic modulation of the Fermi level of graphene with respect to its Dirac point, whereas the smaller negative dipole in mixed 16MHDA + C12 SAM induces weaker modulation of the Fermi level of graphene, as shown in fig. S5 (*45*). A reduced modulation of the Fermi level in graphene within mixed 16MHDA + C12 SAM correlates with a decreased concentration of charge carriers at the Fermi surface, thereby enhancing the electrical transparency of the graphene (i.e., reducing the screening effect of the electric field). The degree of molecular orbital modulation results from the electric field penetrating the graphene, which leads to mixed 16MHDA + C12 SAMs having higher gating efficiency.

In addition, the switching behavior of mixed SAM transistors in response to gate voltages was examined through pulse tests in Fig. 4F. The mixed SAM molecular transistor switches between direct and F-N tunneling transports, leading to a variation in current up to two orders of magnitude. This result is attributed to high drain voltage tolerance and enhanced gating efficiency, leading to an expanded transmission window and greater modulation of molecular orbital levels. These findings indicate that the mixed SAM devices offer an effective molecular transistor platform, capable of probing and using molecules with deep orbital levels in transistor applications.

## DISCUSSION

In our study, we fabricated and characterized the vertical molecular transistor devices made with single-type and mixed SAMs of alkanethiol-based molecules with deep orbital levels. The mixed SAM molecular devices exhibited increased robustness by withstanding higher voltages compared to single-type SAM devices. The current modulation in the molecular transistors was observed to be more pronounced at positive drain voltages, which is attributed to the molecular orbital level entering transmission window. This phenomenon was studied in detail through TVS, which elucidated the different tunneling mechanisms at different drain and gate voltages. We also observed that the mixed SAM transistors showed higher gating efficiency compared with single-type SAM devices due to the suppressed screening effect in graphene by the net molecular dipole. These improvements are crucial for expanding the transmission window and achieving greater modulation of molecular orbital levels. The results of this study demonstrate the mixed SAM molecular transistor platform as a promising approach for developing stable and efficient molecular device applications.

## MATERIALS AND METHODS

### Sample preparation

Silicon substrate was first rinsed in acetone, 2-propanol, and deionized water for 10 min in each step. Electron beam evaporator was used to pattern 50-nm-thick Au on the silicon substrate that is used as the drain electrode. Optical adhesive (OA) was applied on the Au/silicon substrate to undergo template-stripping procedure. Subsequently, ultraviolet (UV)–treated glass was placed onto the substrate and another UV treatment was underwent for 1 hour to harden the OA. After the UV treatment, glass was stripped from the silicon substrate and Au/OA/glass structure was obtained. Then, PR was spin-coated onto the Au/OA/glass sample and photolithography was done to define 2-μm radius holes to expose Au surface. The sample was then annealed at 250°C for 10 min for hard baking of the PR. To form mixed SAMs in the holes, the ReSEM procedure was used (*29*, *30*). After the SAM process, monolayer graphene was wet-transferred onto the sample, which serves as the top electrode. Ion gel (EMIM-TFSI) was deposited on top of the graphene electrode, with the detailed deposition process provided in section S1.2. This step finalizes the molecular transistor device structure.

### Energy level characterization

Energy band characterization of single-type SAM and mixed SAM molecules were conducted with UPS in ESCA Axis Supra+ equipment. For this purpose, molecular SAM was formed on Au film that covers the entire surface of template-stripped glass substrate. To form single-type 16MHDA SAMs, the sample substrates were immersed in solution of 1 mM of 16MHDA for 18 hours, while mixed SAMs were formed by immersion of samples in solution of 1 mM of 16MHDA and C12 through the ReSEM procedure.

KPFM measurements were conducted with NX-10 AFM to determine the effect of molecular dipole on Au. The work functions of the samples were determined with comparison to the reference value (4.475 eV) of highly ordered pyrolytic graphite (*46*).

### Density functional calculations

We carried out equilibrium DFT and nonequilibrium MS-DFT calculations (*40*–*43*) using the SIESTA package (*47*). Double ζ-plus-polarization-level numerical atomic orbital basis sets and a mesh cutoff of 200 Ry for the real-space integration were used together with the Perdew-Burke-Ernzerhof generalized gradient approximation (*48*) and the Troullier-Martins–type norm-conserving pseudopotentials (*49*). To model the Au-molecule-graphene junctions, we adopted a 4-by-4 Au(111) supercell and a gate electrode modeled by an Au(111) monolayer was placed at 2 nm above the graphene top electrode. A 15-by-15 Monkhorst-Pack k-points in the Brillouin zone were sampled along the surface-parallel direction. The atomic geometries were optimized in equilibrium until the total residual forces on each atom were below 0.02 eV/Å.

## Supplementary Materials

### This PDF file includes:

Supplementary Text Table S1 Figs. S1 to S23 References

Correction (11 July 2025):
In fig. S17B, the less-than sign (<) has been corrected to a greater-than sign (>), and in fig. S17C, the greater-than sign (>) has been corrected to a less-than sign (<). In the Supplementary Text, the original sentence “For positive gate voltages (Figure S18B), graphene becomes p-doped, whereas for negative gate voltages (Figure S18C), it becomes n-doped” has been corrected to “For negative gate voltages (Figure S18B), graphene becomes p-doped, whereas for positive gate voltages (Figure S18C), it becomes n-doped.” The Supplementary Materials PDF has been updated. The original version is available here:
