# Supplementary material for: Enhanced gating efficiency in vertical mixed molecular transistors with deep orbital level
Source: Sci Adv. 2025 Jun 18;11(25):eadt3603. doi: 10.1126/sciadv.adt3603 (PMC12175899; doi:10.1126/sciadv.adt3603)
Supplement: Supplementary file 2 — Correction (11 July 2025): In fig. S17B, the less-than sign (<) has been corrected to a greater-than sign (>), and in fig. S17C, the greater-than sign (>) has been corrected to a less-than sign (<). In the Supplementary Text, the original sentence “For positive gate voltages (Figure S18B), graphene becomes p-doped, whereas for negative gate voltages (Figure S18C), it becomes n-doped” has been corrected to “For negative gate voltages (Figure S18B), graphene becomes p-doped, whereas for positive gate voltages (Figure S18C), it becomes n-doped.” The Supplementary Materials PDF has been updated. The original version is available here: [file sciadv.adt3603_sm.v1.pdf]

Science Advances
AAAS

Supplementary Materials for

Enhanced gating efficiency in vertical mixed molecular transistors with deep orbital level

Donguk Kim *et al.*

Corresponding author: Takhee Lee, tlee@snu.ac.kr; Keehoon Kang, keehoon.kang@snu.ac.kr; Yong-Hoon Kim, y.h.kim@kaist.ac.kr

Sci. Adv. **11**, eadt3603 (2025)
DOI: 10.1126/sciadv.adt3603

This PDF file includes:

Supplementary Text
Table S1
Figs. S1 to S23
References

Table S1. Molecules that have been studied in molecular transistors.

Molecule	Molecular structure	$E_F - E_{HOMO}$ (eV)	Device structure	Gating method	†Gating efficiency	Ref no.
**BDT**	Aromatic	1.15**	Au electro-migration	Dielectric field gating	0.22	12
**ODT**	Aliphatic	1.9**	Au electro-migration	Dielectric field gating	0.25	12
**STPP**	Aromatic	1.3	STM-BJ	Electrochemical gating	0.47[b]*	14
**OSTPP**	Aromatic	1.25	STM-BJ	Electrochemical gating	0.11[b]*	14
**Pyridine nitrogen**	Aromatic	0.62	STM-BJ	Electrochemical gating	–	15
**6V6**	Aromatic	0.03	STM-BJ	Electrochemical gating	–	24
**TPCO**	Aromatic	1.4	STM-BJ	Electrochemical gating	–	25
**Ru-DAE**	Aromatic	0.2	Graphene nanogap	Dielectric field gating	0.07[a]*	13
**Biphenyl**	Aromatic	0.75	Graphene nanogap	Electrochemical gating	0.2[e]*	26
**Triphenyl**	Aromatic	0.62	Graphene nanogap	Electrochemical gating	0.36[e]*	26
**Hexaphenyl**	Aromatic	0.9	Graphene nanogap	Electrochemical gating	0.42[e]*	26
**OAE**	Aromatic	1.58	Vertical large-area	Ionic electrostatic gating	–	17
**Anthanthrene**	Aromatic	0.8	Vertical large-area	Ionic electrostatic gating	0.12[d]	18
**OPE3**	Aromatic	1.03	Vertical large-area	Ionic electrostatic gating	0.075[c]	16
**PCP**	Aromatic	1.03	Vertical large-area	Ionic electrostatic gating	0.19[c]	16
**16MHDA**	Aliphatic	2	Vertical large-area	Ionic electrostatic gating	0.24	***This work***

*Obtained from theoretical calculations

[†]Gating efficiency estimated from DFT transmission curves or dJ/dV versus V_D characteristics

[**]Obtained from V_{trans} values in Fig. 1c, 2c (with extrapolation)

[a]Evaluated from Fig. 4a

[b]Evaluated from Fig. 4c, 4d

[c]Evaluated from Fig. 4b, 4e

[d]Evaluated from Fig. 4b

[e]Evaluated from Fig. S15, 4a, 4b

The molecule abbreviations are; benzendithiol for BDT, octanedithiol for ODT, ruthenium-diarylethene for Ru-DAE, 5,15-bis(4-(methylthio)phenyl)porphyrin for STPP, 5,15-bis(4-(methylthio)phenyl)-1H,9H,10H,20H-porphyrin10,20-dione for OSTPP, pseudo-p-bis((4-(acetylthio)phenyl)ethynyl)- p-[2,2]cyclophane for PCP, 1,4-bis(((4-acetylthio)phenyl)ethynyl)benzene for OPE3, N,N'-Di-(6-(thioacetyl)hexyl)-4,4'-bipyridinium bis(hexafluorophosphate) for 6V6, thiophene/phenylene co-oligomers for TPCO and 16-mercaptohexadecanoic acid for 16MHDA. Anthanthrene evaluated in the table is for the 7, 2` connectivity. TPCO evaluated in the table is for the S-TTT-S configuration. STM-BJ stands for scanning tunneling microscope-break junction.

In Table S1, the ionic electrostatic gating employed in these large-area molecular transistors demonstrates the highest reported gating efficiency. Notably, this value remains comparable to that achieved through dielectric field gating. It is worth emphasizing that this enhanced gating efficiency was obtained using an aliphatic molecule possessing a deep molecular orbital level.

1. Device fabrication

1.1 Molecular transistor device fabrication process

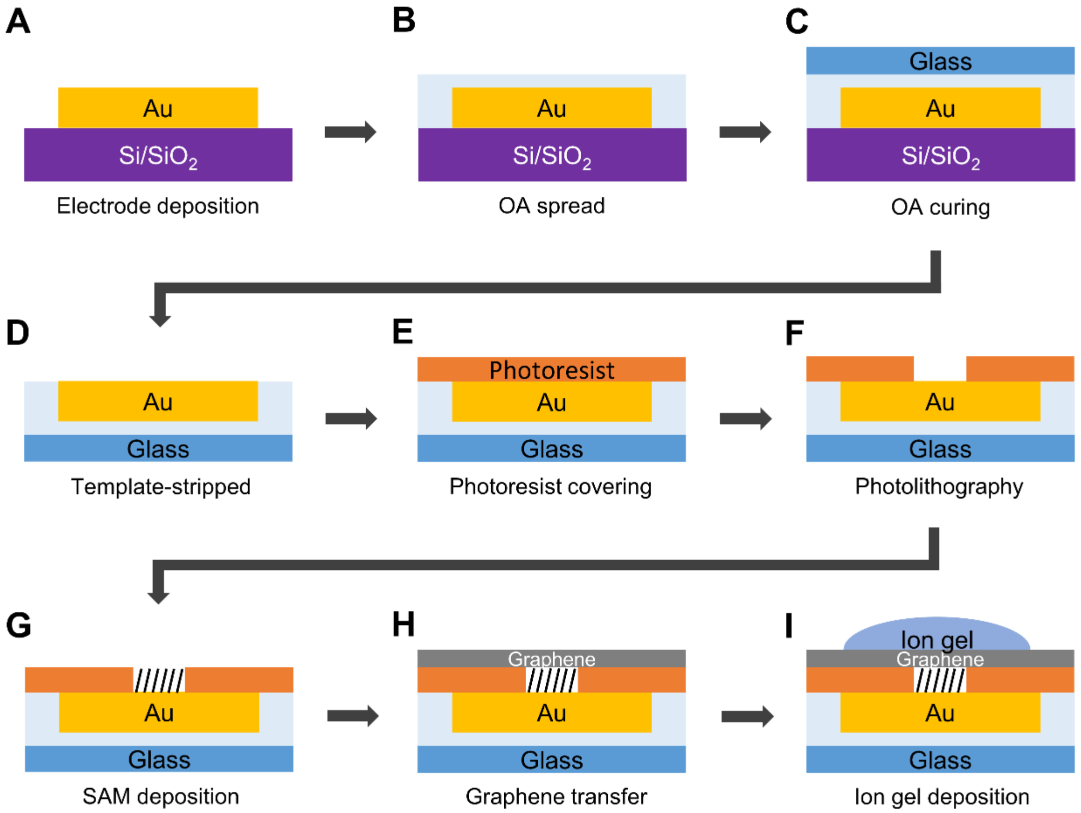

Figure S1. Fabrication method of molecular transistors. Schematics illustrating the step-by-step process of device fabrication. (**A**) Deposition of Au on Si substrate. (**B**) Spreading optical adhesive (OA) on the sample. (**C**) Curing OA and covering glass on the sample. (**D**) Peeling off Au from Si substrate. (**E**) Spin coating of photoresist. (**F**) Patterning photoresist to expose Au surface. (**G**) SAM deposition. (**H**) Wet-transfer of graphene. (**I**) Deposition of ion gel.

1.2 Ion gel deposition

Figure S2 illustrates the deposition process of ion gel. The deposition of the ion gel was accomplished with the use of thin masking tape. The masking tape was punctured in the middle allowing for the exposure of the gate electrode and holes with molecular SAMs. EMIM-TFSI was used as the ion gel in this study, with PS-PMMA-PS as the polymer and ethyl acetate as the solvent. Ion gel was deposited within the puncture masking tape hole and put in vacuum overnight. After which the masking tape was subsequently removed, leaving only the vacuum dried ion gel fixated on the gate electrode and holes.

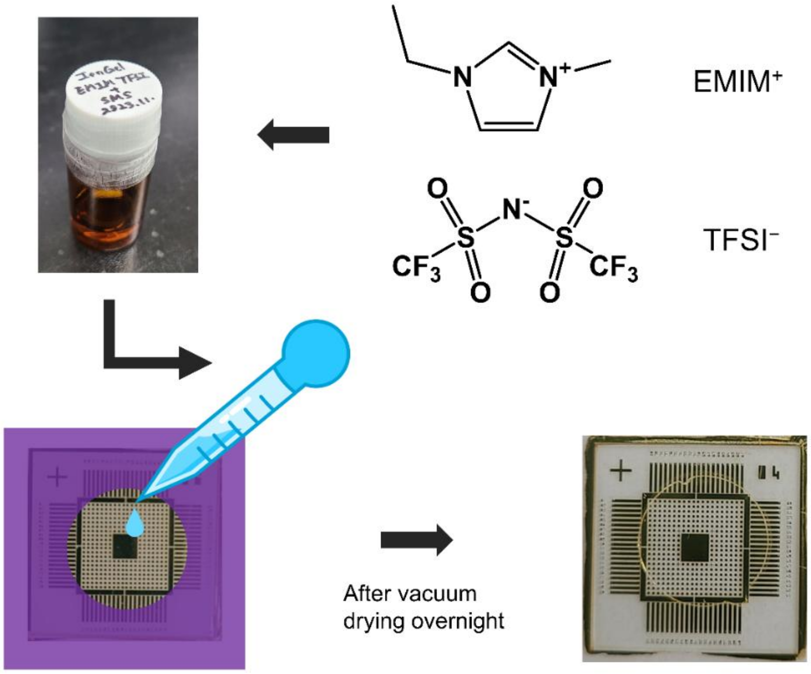

Figure S2. Deposition process of ion gel

2. Electrode and junction characteristics

2.1 Au morphology measurements

Figure S3 shows comparison of surface roughness of Au substrates that were prepared with and without template-stripping (TS) procedure. Au substrate that is prepared by thermal or electron beam evaporation has root-mean-square (rms) surface roughness of around 1.3 nm which is similar to the order to the length of molecules in this study (approximately 2 nm). This often result in electrical short problem, as the probability of the Au bottom electrode directly contacting the graphene top electrode increases. To prevent this problem, we used the template-stripping (TS) method to flatten the Au substrate and reduce its surface roughness (*50*). A glass substrate is covered on the evaporated Au on Si substrate using an optical adhesive. After ultraviolet treatment, Si substrate is carefully removed, leaving the Au-glass substrate. As the exposed part of the Au substrate is the side where it was in contact with the ultra-flat Si substrate, the Au substrate is flattened with rms surface roughness (~0.4 nm).

Figures S3A and S3C show AFM image and histogram of surface height of Au substrate prepared without TS procedure. The rms surface roughness of Au substrate prepared without TS procedure is found to be ~1.3 nm (Fig. S3C). Figures S3B and S3D show AFM image and histogram of surface height of Au substrate prepared with TS procedure. The rms surface roughness of Au substrate prepared with TS procedure is found to be 0.4 nm (Fig. S3D). From the surface roughness improvement in Au substrates through the TS procedure, we have confirmed that this procedure leads to flatter Au surfaces.

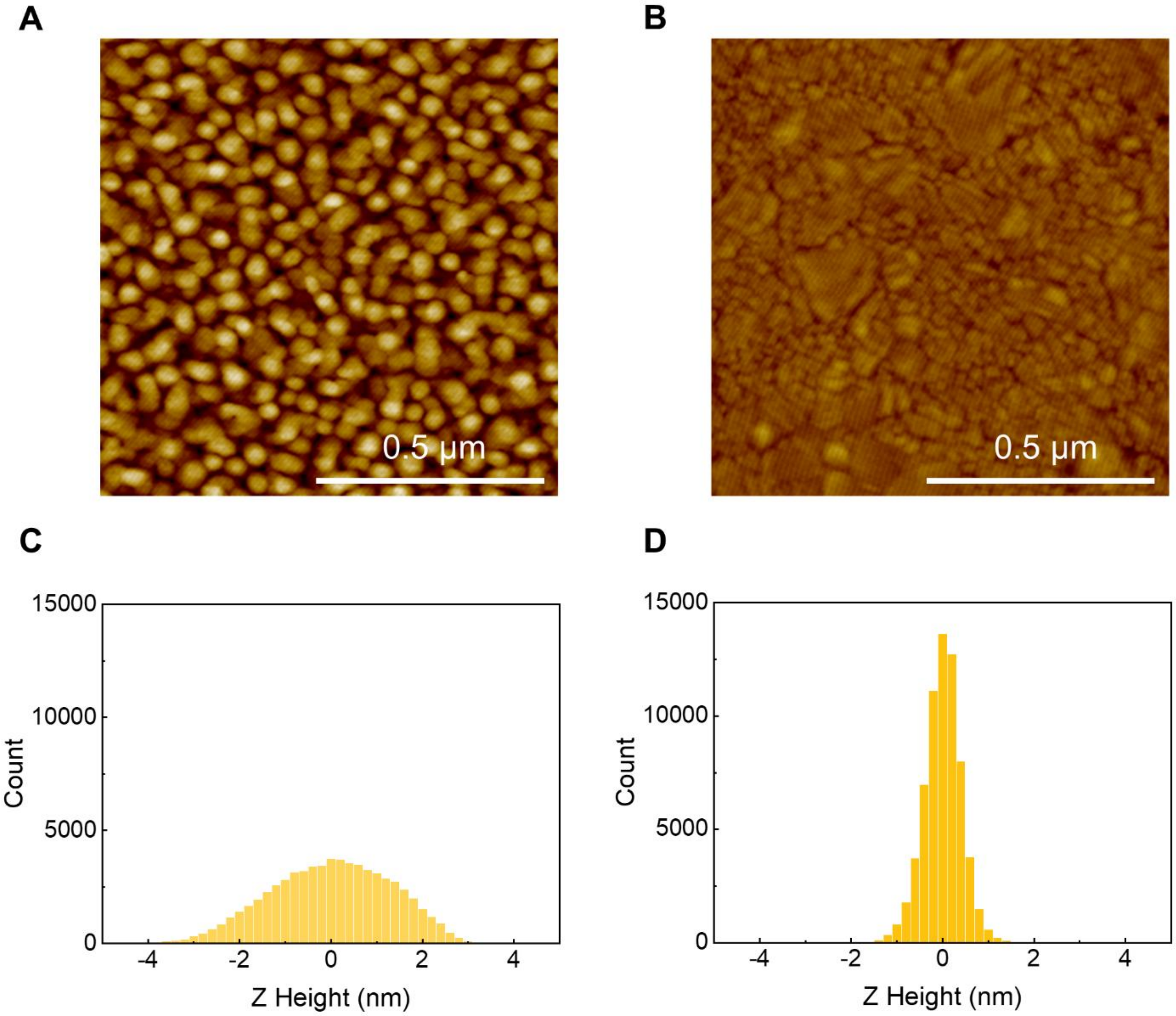

Figure S3. Au morphology characteristics. (**A,B**) AFM images of Au substrates prepared (**A**) with and (**B**) without TS method. (**C,D**) Histograms of the surface height of Au substrates prepared (**C**) with and (**D**) without TS method.

2.2 KPFM measurements of molecules/Au samples

KPFM measurements reveal the change of work function of Au substrates deposited with molecular SAMs. In order to obtain accurate work function values in KPFM measurements, the work function offset (Φ_t) must be determined. The equation $\Phi_t = \Phi_s + eV_{CPD}$ is used to calculate the work function offset value, where Φ_s is the known work function of the reference sample (highly ordered pyrolytic graphite (HOPG) in our study) and V_{CPD} is the measured surface potential value of the reference sample. The HOPG work function value of 4.47 eV was used in our study and surface potential was measured to be -0.09 V. This gives the work function offset Φ_t of 4.38 eV.

Figure S4 shows schematics of molecular dipoles and corresponding work function change of Au. For C12 molecules/Au, the dipole direction is positive (away from Au), whereas the dipole direction is negative (towards Au) for 16MHDA molecules/Au. The net dipole effect from mixed 16MHDA+C12 molecules/Au creates a small negative dipole. The vacuum level modulation that leads to the change of work function of Au was characterized by KPFM measurements and the resulting values are presented in Fig. S4 (also see Fig. 1D in the main manuscript).

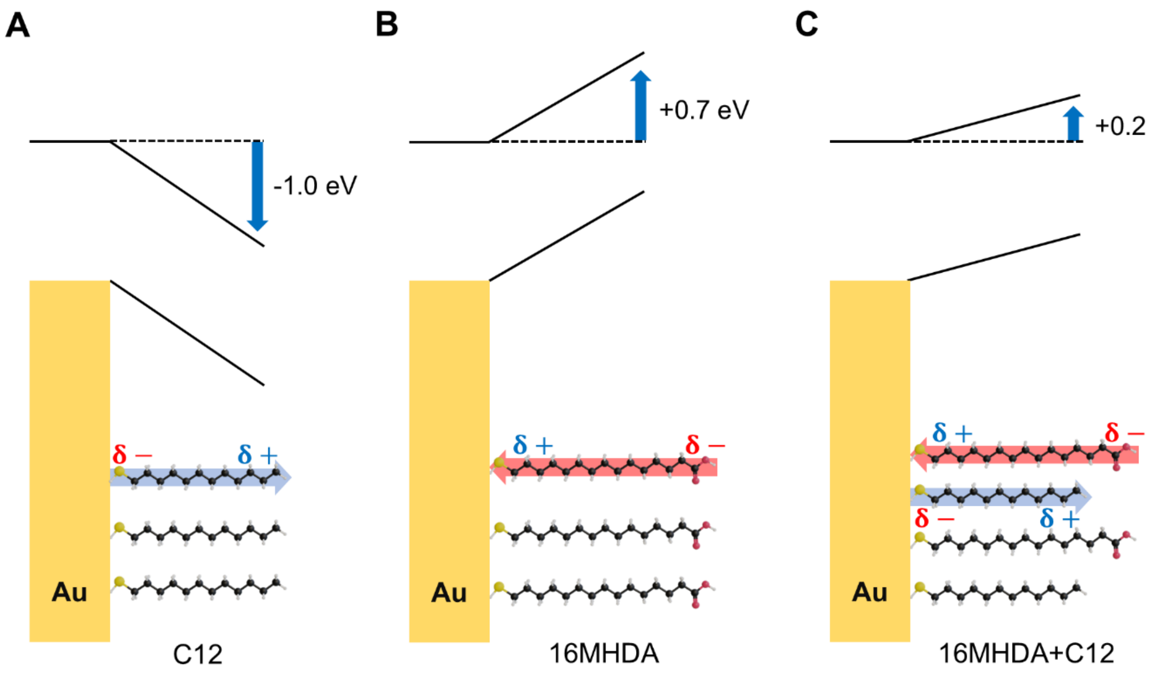

Figure S4. Schematics of molecular dipoles and work function change of Au. Molecular dipoles of (**A**) C12/Au, (**B**) 16MHDA/Au, and (**C**) 16MHDA+C12/Au. Corresponding vacuum levels are shown above.

To study the effect of molecular dipole on graphene work function, KPFM measurements with Au/molecule/graphene structure was conducted, as shown in Fig. S5. When graphene was on 16MHDA SAMs, the average measured surface potential value was found to be -0.37 V. This value changed to the average of -0.19 V when graphene was on 16MHDA+C12 SAMs. From the work function offset (Φ_t) value of 4.38 eV, the work function of graphene on 16MHDA SAMs was determined to be 4.75 eV while the work function of graphene on 16MHDA+C12 SAMs was determined to be 4.57 eV.

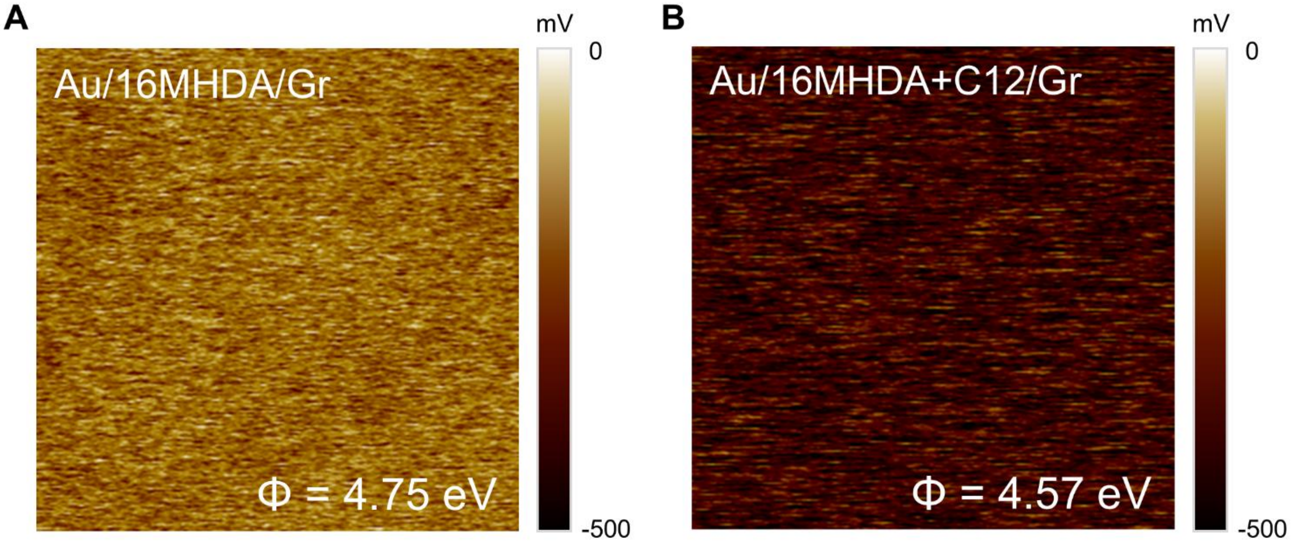

Figure S5. Graphene work function modulated by molecular dipoles. Work function measurements of (**A**) Au/16MHDA/Gr, (**B**) Au/16MHDA+C12/Gr.

2.3 Raman spectroscopy of graphene electrode

Raman spectroscopy was performed on the graphene used in this study to confirm its monolayer quality. The results, presented in Figure S6, reveal distinct peaks characteristic of graphene. The G peak, corresponding to in-plane vibrations of sp^2-hybridized carbon atoms, and the 2D peak, arising from a second-order two-phonon scattering process in the absence of defects, are clearly observed. The prominent and characteristic profiles of these peaks confirm that the graphene used in this study is a monolayer.

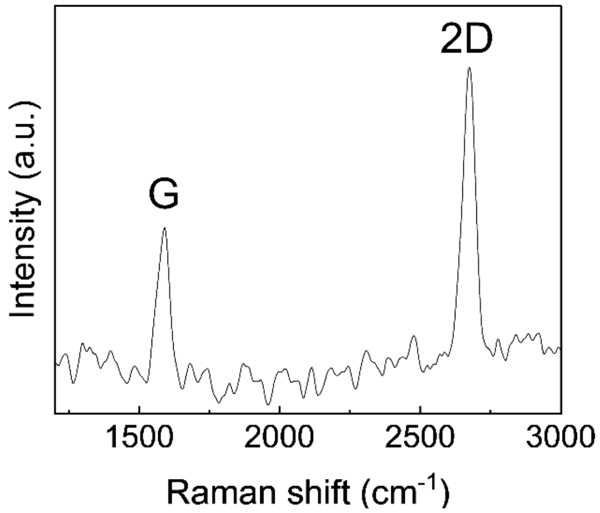

Figure S6. Raman spectroscopy of monolayer graphene. Plot shows two prominent peaks labeled as G and 2D peaks.

3. Work function modulation of dipoles

3.1 Cyclic voltammetry

To corroborate our work function modulation values, we conducted dipole moment calculations and cyclic voltammetry. Performing molecular density functional theory (DFT) calculations with the B3LYP functional and the Def2-TZVP basis set, we obtained for 16MHDA a dipole moment of 1.36 D. On the other hand, C12 displayed a dipole moment of -0.84 D, confirming the presence of oppositely oriented dipoles for the two molecules. Figure S7 shows the cyclic voltammetry curves which were conducted to quantify surface coverage. The experimental setup included a SAM-coated Au working electrode (1 cm^2 area), an Ag/Ag^+ reference electrode containing 0.1 M TBAP and 0.01 M $AgNO_3$ in MeCN, a Pt coil counter electrode (2.51 cm^2 active area), and 0.1 M BMP:TFSI in MeCN as the electrolyte. Surface coverage was calculated using the equation $\Gamma = 4RTi_{pa}/n^2F^2A\upsilon$, where R is the gas constant (8.314 $J{\cdot}mol^{-1}{\cdot}K^{-1}$), T is the temperature (298.15 K), n is the number of electrons transferred per reaction (assumed as 1), F is the Faraday constant (96485 $C{\cdot}mol^{-1}$), i_{pa} is the anodic peak current, A is the working electrode area (1 cm^2), and υ is the scan rate. Anodic peaks were used for fitting the cyclic voltammetry data.

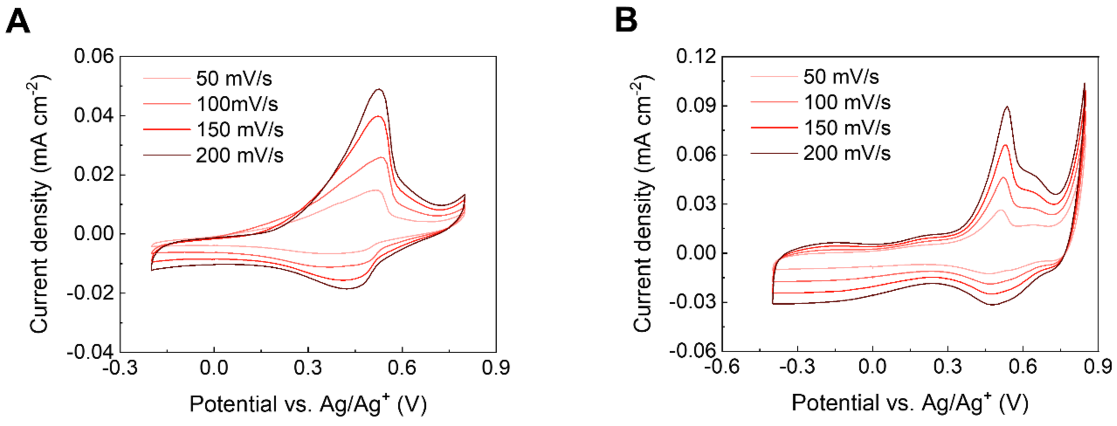

Figure S7. Cyclic voltammetry curves. Plots are shown for (**A**) 16MHDA and (**B**) C12 molecules.

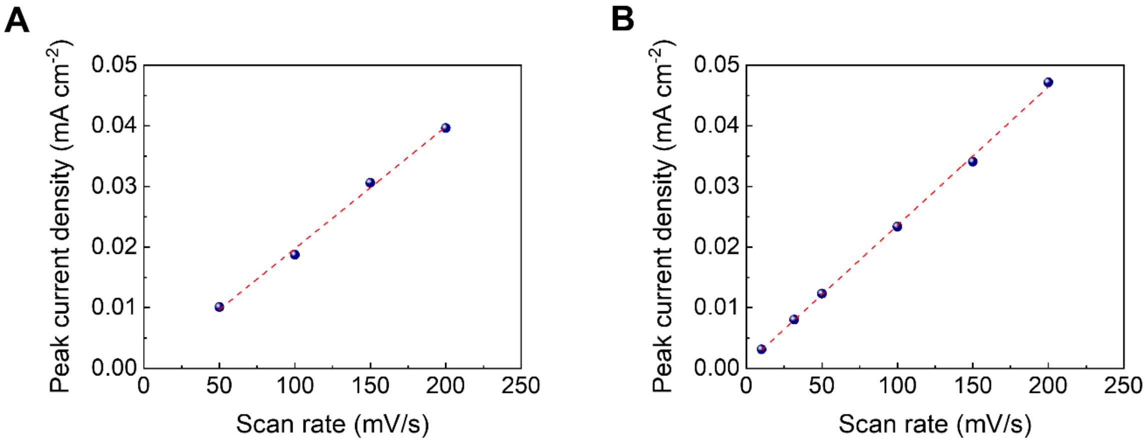

Figure S8. Scan rate dependency of voltammograms. Plots are shown for (**A**) 16MHDA and (**B**) C12 molecules.

The scan rate dependence of the voltammograms, shown in Figure S8, demonstrates a linear correlation between i_{pa} and υ for both 16MHDA and C12 molecules at scan rates ≤ 200 mV/s. This behavior aligns with the equation $i_{pa} = n^2F^2\mathrm{A}\Gamma\upsilon/4\mathrm{RT}$, validating the surface coverage model within this range. The calculated surface coverage values were $2.11 \pm 0.07 \times 10^{-10}$ mol·cm^{-2} for 16MHDA and $2.51 \pm 0.07 \times 10^{-10}$ mol·cm^{-2} for C12. These values, combined with the calculated dipole moments, resulted in work function modulations of +0.65 eV for 16MHDA and -0.47 eV for C12. These findings are consistent with the KPFM measurements performed in this study and align with the expected modulation directions.

3.2 DFT calculations

We additionally carried out DFT calculations for Au-SAM heterostructures within the periodic boundary condition and show in Figure S9 plane-averaged electrostatic potentials. The x-axis (Distance) in this figure indicates the distance from the Au slab. The calculated work function of 5.10 eV for the pristine Au(111) surface is consistent with the experimental value. With respect to this value, we obtained shifts of -0.49 eV and +0.87 eV with C12 and 16MHDA adsorptions, respectively. These work function modulations are primarily governed by the terminal functional groups of C12 and 16MHDA: With the electron-donating methylene group (-CH_2), electrons transfer from C12 to Au(111) and the work function decreases. In contrast, the electronegative carboxyl group (-COOH) withdraws electrons from the Au surface, resulting in an increase of the work function.

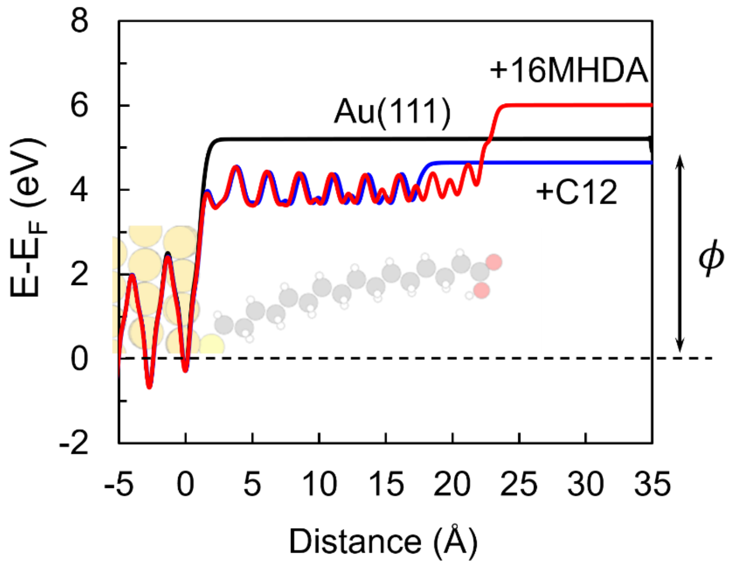

Figure S9. DFT-derived plane-averaged electrostatic potentials. The electrostatic potentials are shown for pristine Au(111) slab (black), C12-adsorbed Au slab (blue), and 16MHDA-adsorbed Au slab (red).

4. Electrical measurements

4.1 Electrical characteristics of molecular junctions and breakdown phenomenon

Figure S10A shows the electrical characteristics of C12 and 16MHDA molecular junctions. The current of C12 is larger than that for 16MHDA junctions due to the smaller molecular length of C12. C12 molecular length is 1.6 nm and 16MHDA molecular length is 2.1 nm. From our measurement, the current is μA range for C12 and 0.1 μA range for 16MHDA at 1.8 V (Fig. S10A).

Figure S10B shows the breakdown voltage phenomenon of 16MHDA and mixed 16MHDA+C12 molecular junctions. Breakdown occurs at which point the current increases abruptly by several orders of magnitude. The breakdown voltages are ~1.3 V and ~2.4 V for 16MHDA and 16MHDA+C12 junctions, respectively. Several factors may contribute to the breakdown phenomenon, for example, defects within the SAMs that facilitate the migration of metallic atoms, leading to the formation of short circuits (*51*). In mixed 16MHDA and C12 junctions, the breakdown can be mitigated by the increased packing density resulting from the reduced gauche defect junction due to C12 reinforcement molecules and the increased dimerization of -COOH moieties of the 16MHDA matrix molecules (*30*).

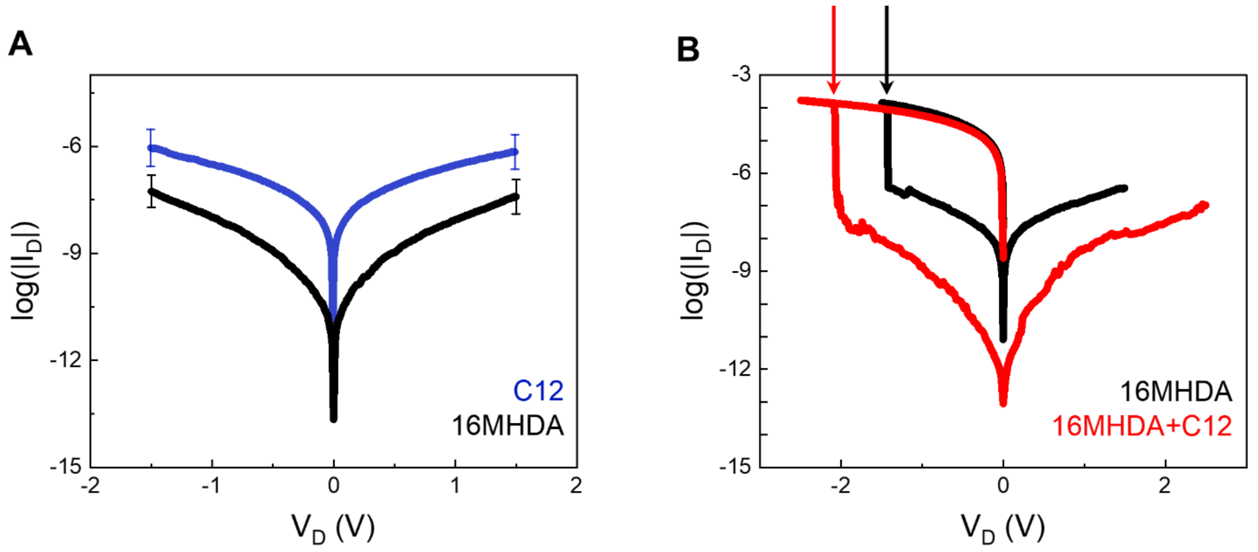

Figure S10. Electrical characteristics. (**A**) Current-voltage characteristics of 16MHDA and C12 molecular junctions. (**B**) Breakdown phenomenon of 16MHDA and mixed 16MHDA+C12 molecular junctions. Arrows indicate the breakdown voltages.

4.2 Histograms of currents of molecular junctions

Figure S11 shows histograms of the current in 16MHDA and 16MHDA+C12 molecular junctions, measured at drain voltage of 1.5 V. The average of current is ~0.3 × 10^{-7} A for both 16MHDA and 16MHDA+C12 junctions.

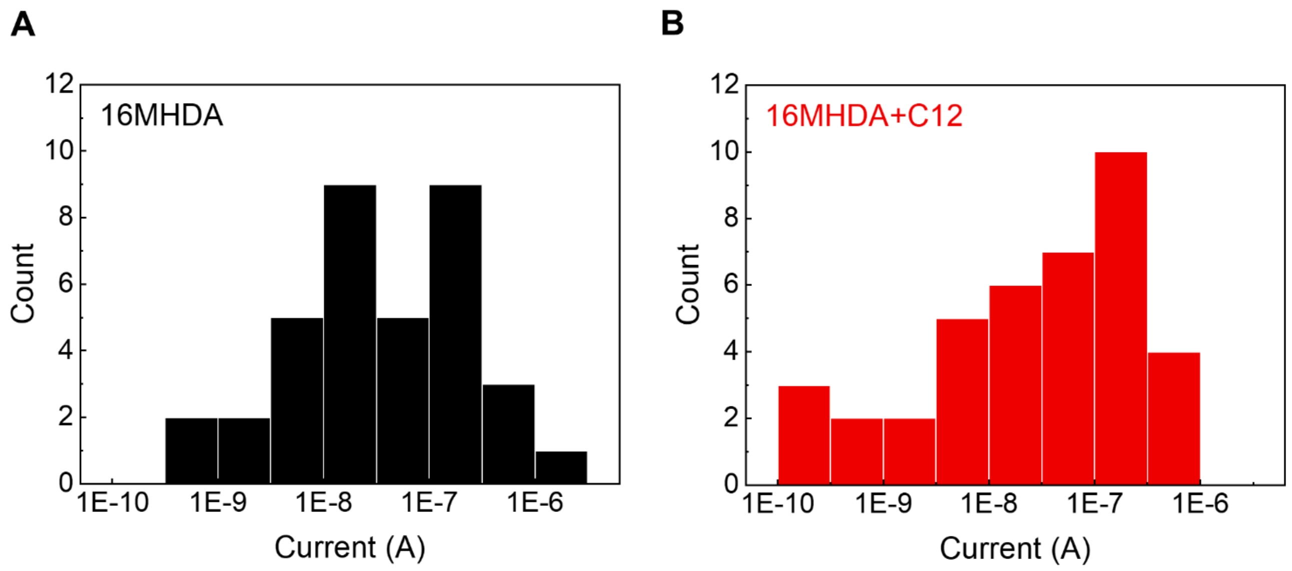

Figure S11. Histograms of currents of molecular junctions. (**A,B**) Current histograms of (**A**) 16MHDA and (**B**) 16MHDA+C12 molecular junctions.

4.3 Temperature-variable electrical measurements

Figure S12 shows the Arrhenius plots of currents of 16MHDA+C12 molecular junction in the temperature range from 80 to 300 K, measured at different drain voltages (V_D) from -2 to 2 V with a step of 0.2 V. Direct tunneling in these molecular junctions yields temperature-independent currents, expected to appear as constant in Arrhenius plots.

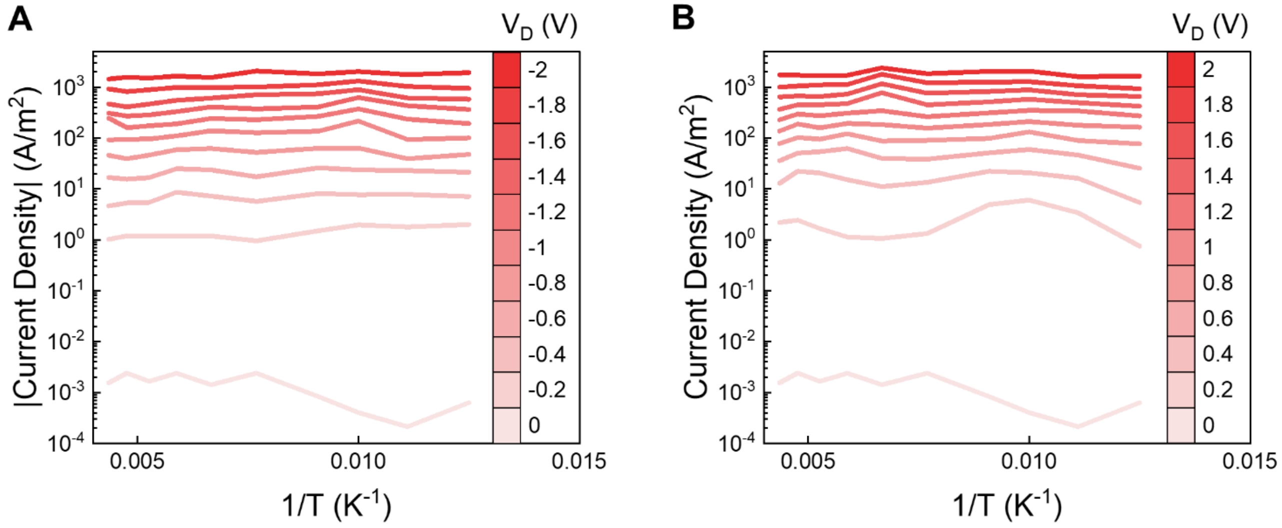

Figure S12. Temperature-variable electrical measurements. (**A,B**) Arrhenius plot of 16MHDA+C12 molecular junction (**A**) under negative and (**B**) positive drain voltages.

5. Ion gel and its effect on molecular devices

5.1 Ion gel

In this study, the ion gel EMIM-TFSI is employed as the gate material. The cations ($EMIM^+$) and anions ($TFSI^-$) differ in size, as illustrated in Figure S13A. The smaller size of the anions results in a higher anion density in the electrical double layer on graphene at negative gate voltages, while the larger cation size reduces the cation density at positive gate voltages (*44*). Consequently, this generates a stronger electric field affecting the molecular orbitals at negative gate voltages, leading to increased gate efficiency under these conditions.

The optical image of the molecular junctions with ion gel is shown in Fig. S13B. The red circles (false color, not to scale) indicate the photoresist holes where SAMs are formed. Ion gel is shown in blue false color, where it extends over the photoresist holes.

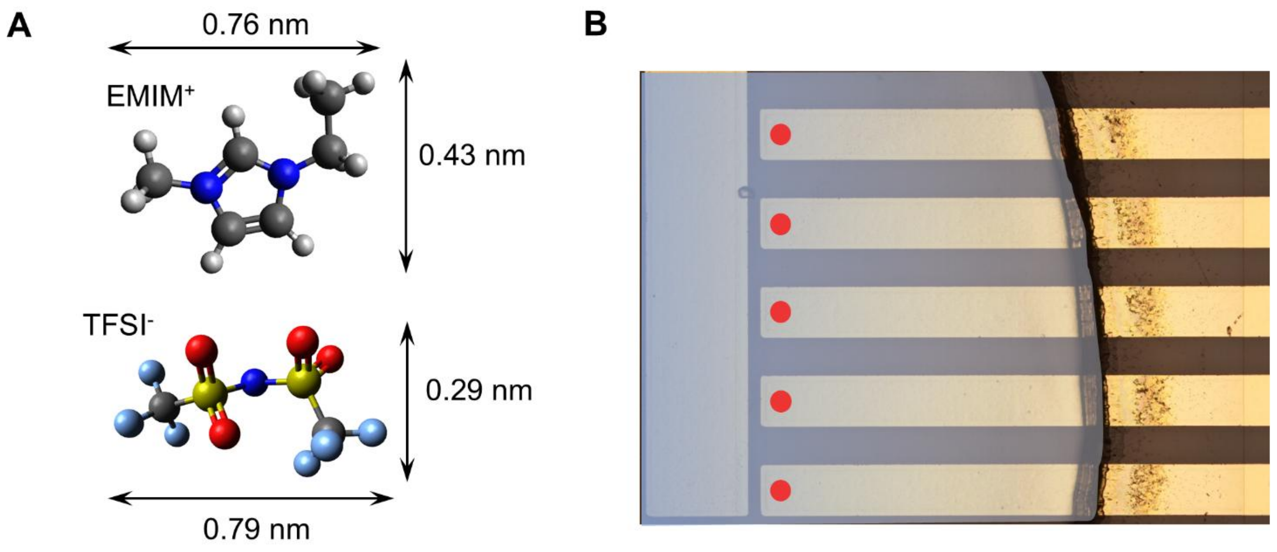

Figure S13. Ion gel. (**A**) Molecular schematics of anions and cations used in this study. Anion $TFSI^-$ is larger in size than cation $EMIM^+$ by 0.1 nm. (**B**) Optical image of the molecular devices with ion gel. Area of ion gel is denoted in false blue color.

5.2 Effect of ion gel on current characteristics of molecular devices

Figure S14A shows current-voltage characteristics of the mixed 16MHDA+C12 devices with and without ion gel. Before ion gel is introduced, no rectification can be seen in the molecular devices, however after ion gel deposition, rectification with larger current at positive voltage can be seen. This behavior is likely due to interfacial modifications at the molecular-graphene and graphene-ion gel junctions following the ion gel application (such as the p-doping effect of graphene) (*52*). Note that the rectification ratio increases as higher drain voltage is applied for the molecular devices with ion gel (Fig. S14B).

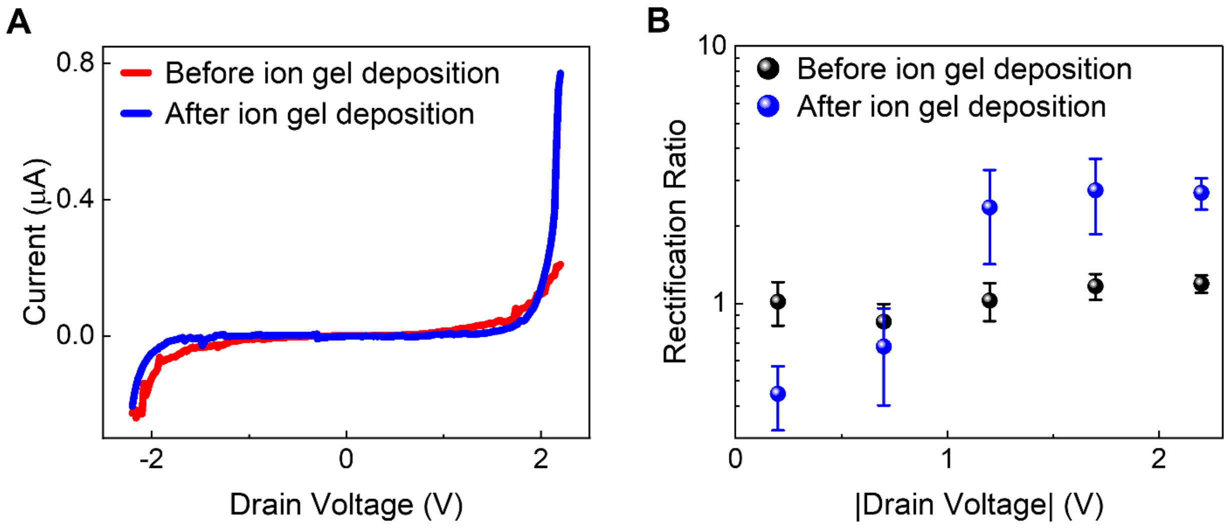

Figure S14. Current characteristics with and without ion gel. (**A**) Current-voltage characteristics of 16MHDA+C12 devices with and without ion gel. (**B**) Rectification ratios of 16MHDA+C12 devices with and without ion gel.

6. Supplementary gate modulation of molecular orbitals

Figure S15A shows the gate modulation of single-type 16MHDA SAM device. As was observed in the gate modulation of mixed 16MHDA+C12 SAM devices, the conductance increases as gate voltage is negatively swept. This confirms that the p-type transistor behavior is consistent in both single-type and mixed SAM devices. Figure S15B shows a comparison between the transfer curves of single-type SAM transistors at +1.3 V and -1.3 V drain voltage biases. In alignment with mixed SAM transistors, the transfer curves under positive drain voltage demonstrated high on/off ratio at ~5, while negative drain voltage transfer curves remained unresponsive.

Figure S16 presents the transfer curves for the 16MHDA+C12 device at drain voltages of +2 V and -2 V. Similar to the behavior observed in the 16MHDA device shown in Fig. S15, the current increases with the application of a negative gate voltage at positive drain voltage, while it remains unresponsive under negative drain voltage.

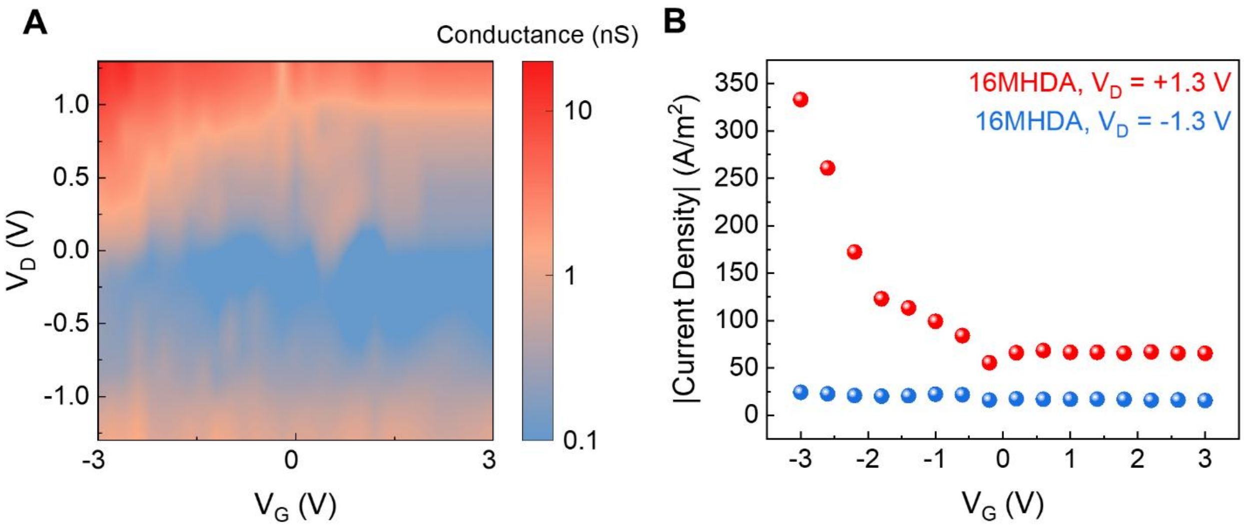

Figure S15. Gate modulation of 16MHDA transistor. (**A**) Contour plot of conductance (dI/dV_D) as functions of V_D and V_G for a 16MHDA transistor. (**B**) Drain current density versus gate voltage at V_D = -1.3 V and +1.3 V for 16MHDA device.

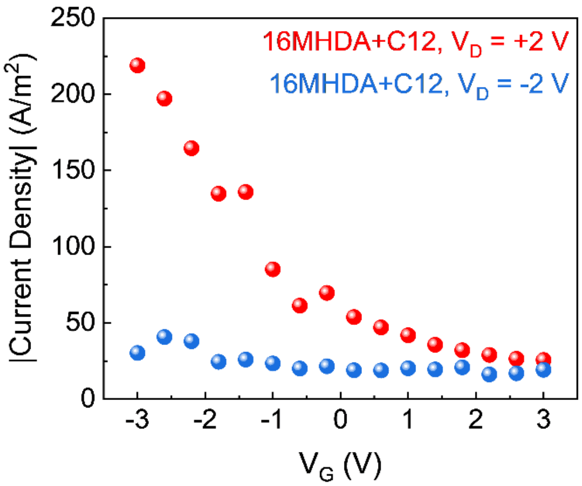

Figure S16. Transfer curve of 16MHDA+C12 transistor. Drain current density versus gate voltage at V_D = -2 V and +2 V for 16MHDA+C12 devices.

7. Molecular orbital modulation mechanism

7.1 Energy band alignment in molecular junctions

The mechanism of molecular transistors is explained and shown in detail. In Figure S17A, all electrodes are grounded, representing the equilibrium state of the system. The Fermi levels are aligned across all the electrodes. Due to graphene's lower work function (4.5 eV) compared to that of Au (5.1 eV), the Dirac point of graphene is offset from the Fermi level, leading to a slight p-doping in graphene. Consequently, graphene’s vacuum level decreases relative to that of Au.

In Figure S17B, a positive voltage is applied to the drain electrode, creating a potential difference between two grounded electrodes (gate and source (graphene) electrodes) and the positively biased drain electrode. This voltage induces an external electric field acting on the graphene layer. If graphene were a conventional metal with a large reservoir of charge carriers, it could fully counteract and screen the external electric field, preventing any penetration of the field. However, because graphene is a semi-metal with a limited density of charge carriers, it cannot completely screen the external field. As a result, while the Fermi level difference between the drain and other electrodes is set at V_D through the drain voltage, this difference does not entirely correspond to the vacuum level difference between the drain and source (graphene) electrodes. The vacuum level difference (V_1 in Figure S17B) between the drain and source (graphene) electrodes is not equal to V_D, instead the presence of an unscreened electric field between the gate and source (graphene) electrodes results in the additional vacuum level difference (V_2 in Figure S17B) between them. Now, the total vacuum level difference ($V_1 + V_2$) between the gate and drain electrodes equals to V_D. However, it is important to note that while the vacuum level difference between the source (graphene) and drain electrodes is not equal to V_D, the Fermi level difference between the two electrodes (source and drain) is equal to V_D.

Additionally, the application of a positive electric field directed from the drain to source electrodes subjects the charges in graphene to the imposed field. Thus, when graphene is grounded, electrons flow into the graphene to maintain its potential at 0 V. This results in a slight n-doping of the graphene to stabilize its potential.

In Figure S17C, the drain electrode is negatively biased, inducing effects opposite to those observed in Fig. S17B. The vacuum level of source (graphene) is elevated but not fully, such that the vacuum level difference between source (graphene) and drain does not equal V_D and instead equals V_1. As observed in Fig. S17B, the unscreened electric field between the gate and the source (graphene) induces a vacuum level difference of V_2, such that $V_1 + V_2$ equals V_D. Unlike the scenario in Fig. S17B, the electric field at source (graphene) is directed from source to drain. As a result, electrons flow out of graphene (or equivalently, holes flow in) to maintain its potential at 0 V, leading to slight p-doping of the graphene.

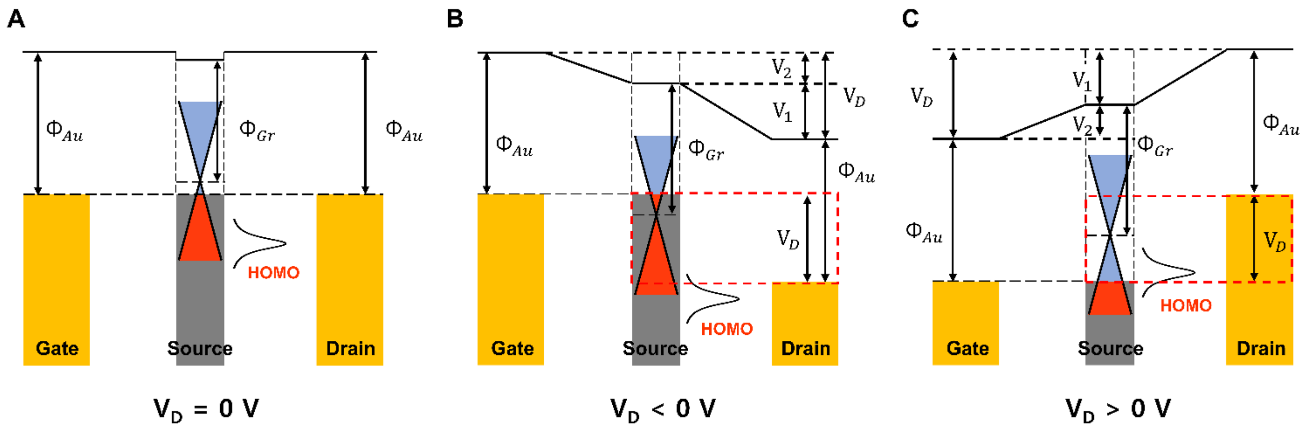

Figure S17. Energy band diagram under drain voltage variation. Energy band diagrams depicting vacuum level and Fermi levels of gate, graphene and drain electrodes. Diagrams represent the cases when (**A**) no drain voltage is applied, (**B**) positive drain voltage is applied and (**C**) negative drain voltage is applied.

Figure S18 below illustrates the energy band diagrams of the Au-molecule-graphene molecular transistor under various gate voltages. In this figure, the Au electrode acts as the drain, the graphene electrode acts as the source, and the Au gate electrode facilitates gating via an ion gel. When no gate voltage is applied, the Fermi levels of the gate, graphene, and drain electrodes are aligned (Figure S18A). However, due to graphene's lower work function (4.5 eV) compared to Au (5.1 eV), a slight p-doping is induced in graphene under equilibrium conditions. Consequently, graphene's vacuum level decreases relative to that of Au.

Upon applying a gate voltage, the Fermi level of the gate electrode shifts relative to those of the source and drain electrodes. The direction of this Fermi level shift depends on the polarity of the applied voltage, for negative gate voltage the Fermi level is elevated (Figure S18B) while for positive gate voltage the Fermi level is lowered (Figure S18C). This creates a voltage difference between the gate and graphene electrodes. However, because graphene is a semi-metal with limited charge carrier density, the electric field is not fully screened, allowing some penetration through the graphene monolayer. As a result, while the Fermi level difference between the gate and other electrodes is set at V_G through the gate voltage, this difference does not entirely correspond to the vacuum level difference between the gate and source (graphene) electrodes. The vacuum level difference (V_1 in Figure S18B) between the gate and source (graphene) electrodes is not equal to V_G, instead the presence of an unscreened electric field between the source (graphene) and drain electrodes results in the additional vacuum level difference (V_2 in Figure S18B) between them. Now, the total vacuum level difference ($V_1 + V_2$) between the gate and drain electrodes equals to V_G.

This incomplete screening also leads to doping effects in graphene. For positive gate voltages (Figure S18B), graphene becomes p-doped, whereas for negative gate voltages (Figure S18C), it becomes n-doped. Additionally, the unscreened electric field between the source and drain electrodes causes a shift in the

molecular orbital energies. Under negative gate voltages, the molecular orbitals shift upward, while under positive gate voltages, they shift downward. This mechanism is central to how the gate modulates the molecular orbitals.

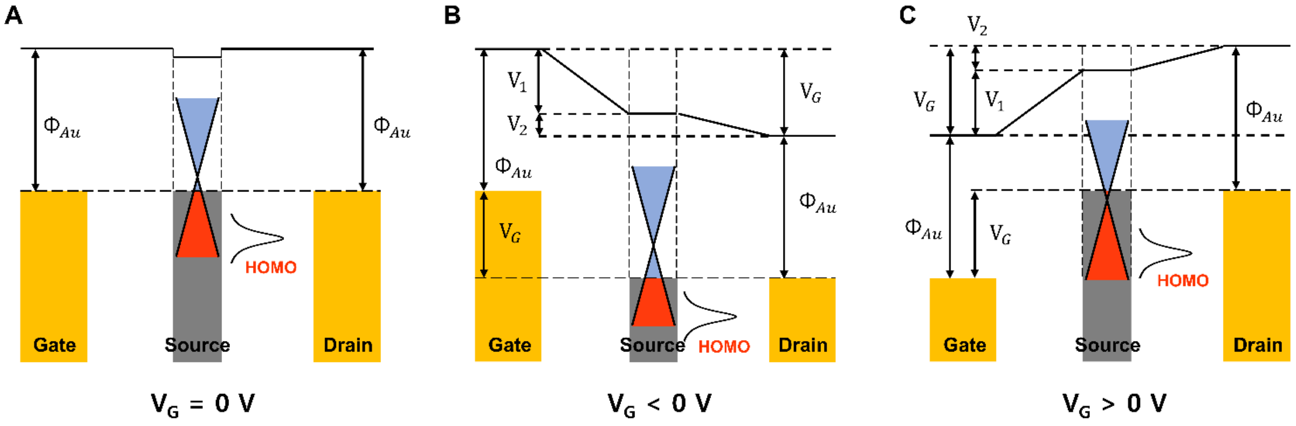

Figure S18. Energy band diagram under gate voltage variation. Energy band diagrams depicting the vacuum level and Fermi levels of gate, graphene, and drain electrodes. Diagrams represent the cases when (**A**) no gate voltage is applied, (**B**) negative gate voltage is applied and (**C**) positive gate voltage is applied.

Figure S19 illustrates both the dipole-induced electric field and the field resulting from the Au–graphene interaction. When Au and graphene are brought into contact, the lower work function of graphene implies that its electrons occupy higher energy states relative to Au. Consequently, electrons transfer from graphene to Au, resulting in p-doped graphene. While this electron transfer lowers the vacuum level of graphene, the corresponding shift in vacuum level of Au is minimal due to its high density of states at the Fermi level. This disparity creates an electric field directed from graphene to Au, which opposes the direction of the dipole-induced field (dipole field along the molecule is directed from Au to graphene). Overall, the net effect is an electric field pointing from graphene to Au. This underlying mechanism explains the Au-molecule-graphene energy level alignment, as well as the work function levels

experimentally observed in graphene over SAMs. This observation is consistent with our previous interpretation that graphene exhibits stronger p-doping when interfaced with the single-type SAM compared to the mixed SAM.

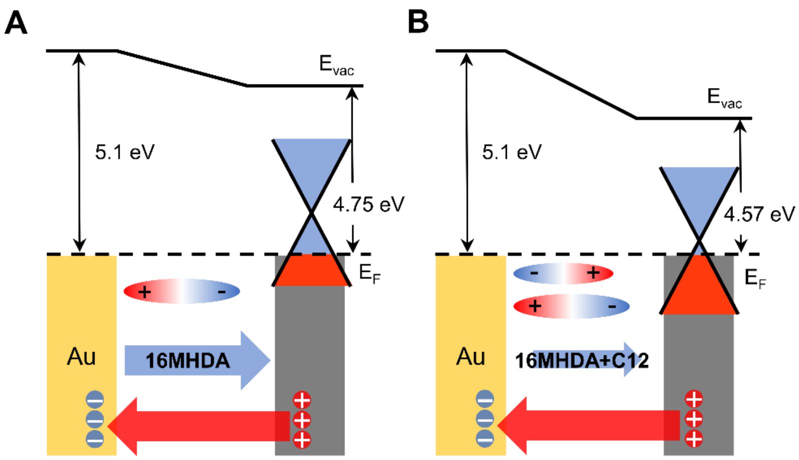

Figure S19. Energy band diagram showing detailed electric field directions. Schematics of (**A**) the 16MHDA molecular junction and (**B**) the mixed 16MHDA+C12 molecular junction.

7.2 First-principles device calculations

To understand the mechanisms of molecular orbital gating, we carried out multi-space constrained-search DFT (MS-DFT) calculations. Computational results shown in Figure S20 align well with the experimental data shown in Figures S15 and S16, indicating that the mechanistic origins of experimentally observed device characteristics are well captured in our simulations. The transfer curves obtained at drain voltages (V_D) of +1 V and -1 V over a gate voltage (V_G) range of +10 V to -10 V reveal that applying a negative V_G with a positive V_D increases the current. On the other hand, under a negative V_D, the current shows only minor variations with changes in V_G. Accordingly, the rectification ratios increase by applying negative V_G. Increasing the magnitudes of V_D to +2 V and -2 V, we find that the trends are overall well maintained but the rectification ratios can be further increased.

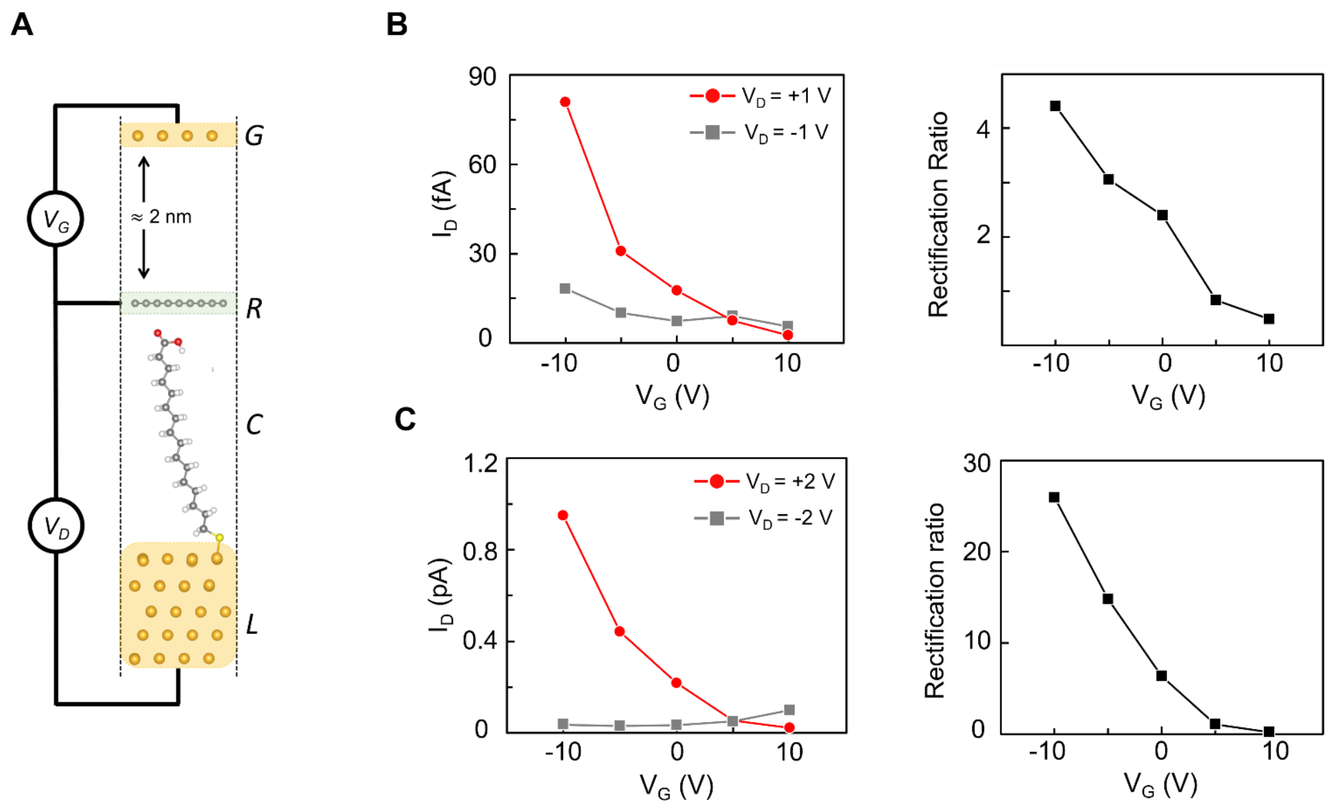

Figure S20. MS-DFT calculation models and results. (**A**) The atomistic junction model. Variations of transfer curves (left panels) and rectification ratios (right panels) as a function of gate voltage at (**B**) V_D = ±1 V and (**C**) V_D = ±2 V.

Based on the nonequilibrium electronic structures, we now discuss the mechanisms of orbital gating in more detail. In Figure S21A, we present the projected density of states (PDOS) of total 9 cases corresponding to the combination of V_G = +10 V, 0 V, and -10 V and V_D = +1 V, 0 V, and -1 V. In Figure S21B, we also present the PDOS for the V_D = +2 V, 0 V, and -2 V cases. Red and blue lines represent the states originating from 16MHDA and graphene, respectively. We find that the transmission within the bias window (not shown) is primarily derived from the highest occupied molecular orbital (HOMO) associated with the carboxyl end groups (indicated by left triangles). As V_G negatively increases, the HOMO level upshifts and moves toward the bias window, increasing the current. Conversely, when a positive V_G is

applied, the HOMO downshifts and moves away from the bias window, reducing the transmission. This trend is particularly enhanced at the positive V_D polarity where the HOMO energetically moves closer to the bias window. Accordingly, the current overall increases in the negative V_G and positive V_D regimes. These orbital gating mechanisms are schematically summarized in Figure 3E.

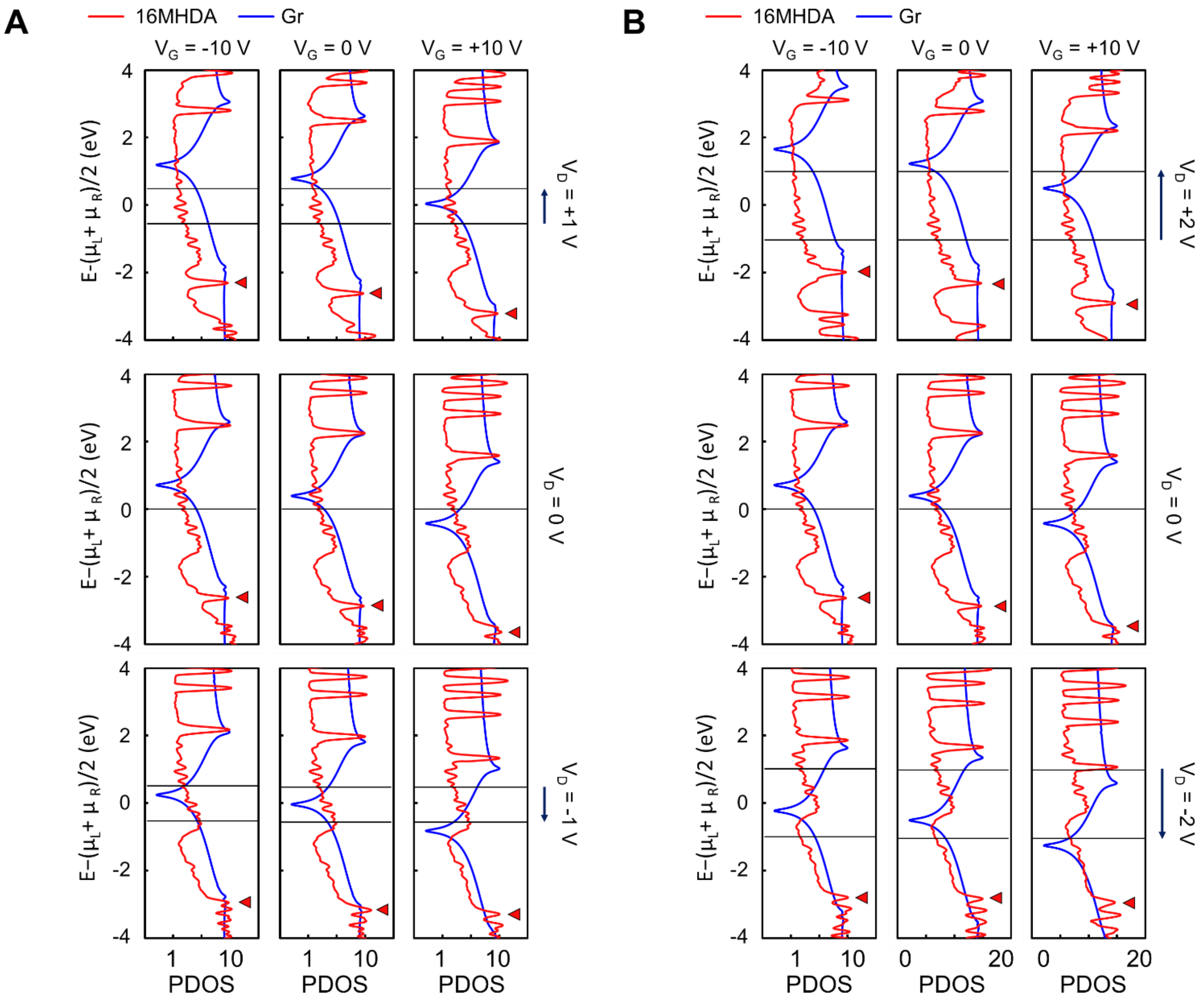

Figure S21. Analysis of the orbital gating mechanism. The PDOS for V_G = -10 V, 0 V, and +10 V (from left to right) are presented for (**A**) V_D = +1 V, 0 V, and -1 V and (**B**) V_D = +2 V, 0 V, and -2 V (from top to bottom). Left triangles indicate the carboxyl group-derived HOMO PDOS peaks.

8. Retention characteristics

8.1 Retention response of molecular transistor

Figure S22 shows the full retention characteristics of 16MHDA+C12 transistor (see also Fig. 4F in the main manuscript). The initial response of the molecular transistor is unstable, which is attributed to the process by which the ion gel is reorganized in response to the gate voltage. After repeated voltage pulses, the molecular transistor returns to stable switching behavior.

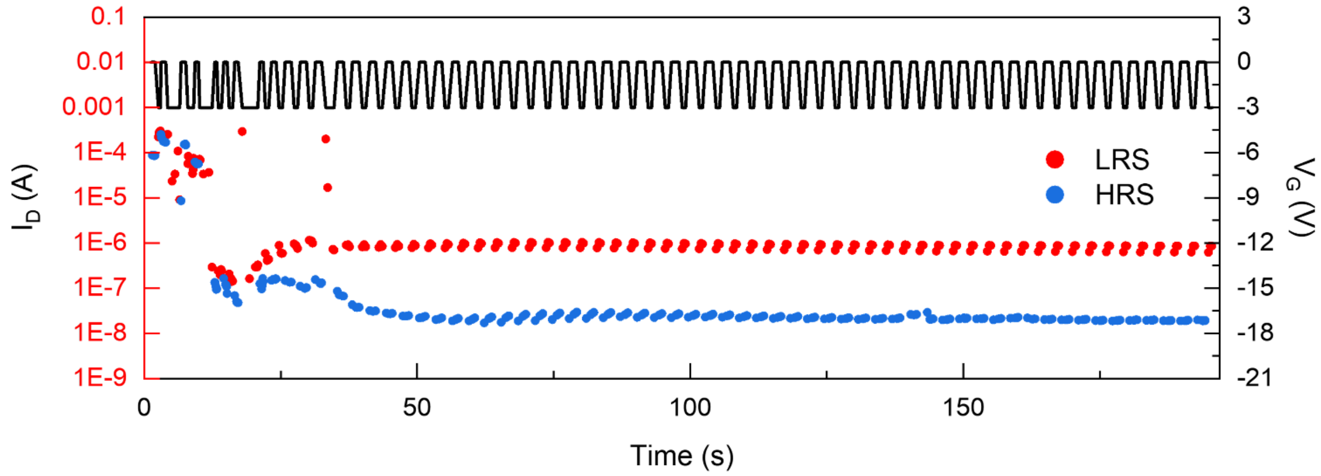

Figure S22. Retention characteristics of 16MHDA+C12 molecular transistor.

Figure S23 shows the switching data of a 16MHDA+C12 transistor after electrical breakdown. The shorted molecular device has smaller on/off ratios that make it difficult to distinguish between high and low resistance states.

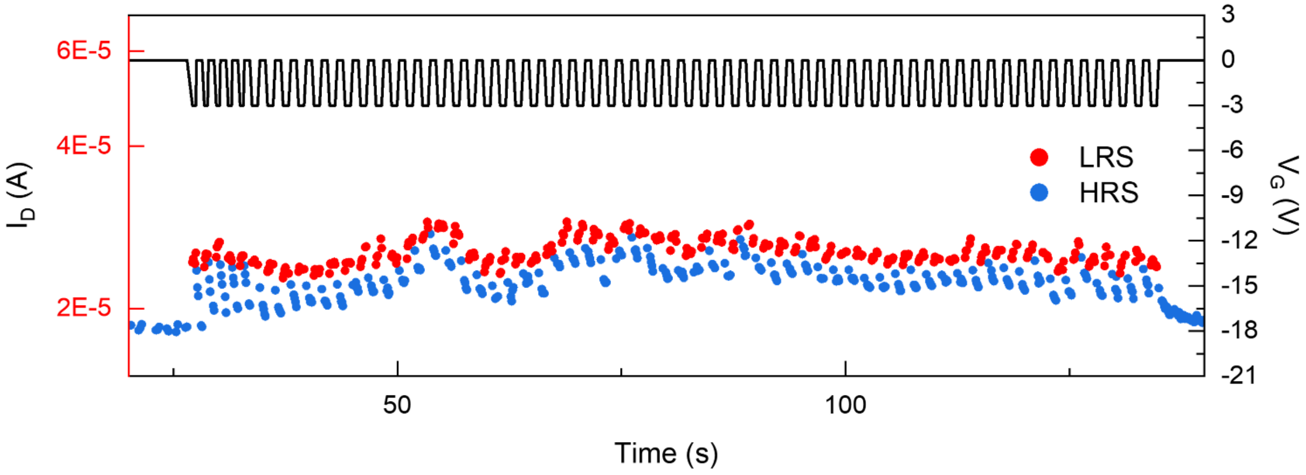

Figure S23. Retention characteristics of an electrically shorted molecular transistor.

REFERENCES AND NOTES

1. A. Aviram, M. A. Ratner, Molecular rectifiers. *Chem. Phys. Lett.* **29**, 277–283 (1974).

2. M. A. Reed, C. Zhou, C. J. Muller, T. P. Burgin, J. M. Tour, Conductance of a molecular junction. *Science* **278**, 252–254 (1997).

3. J. C. Cuevas, E. Scheer, *Molecular Electronics: An Introduction to Theory and Experiment* (World Scientific Publishing, 2010).

4. D. Xiang, X. Wang, C. Jia, T. Lee, X. Guo, Molecular-scale electronics: From concept to function. *Chem. Rev.* **116**, 4318–4440 (2016).

5. M. N. Wang, T. Wang, O. S. Ojambati, T. J. Duffin, K. Kang, T. Lee, E. Scheer, D. Xiang, C. A. Nijhuis, Plasmonic phenomena in molecular junctions: Principles and applications. *Nat. Rev. Chem.* **6**, 681–704 (2022).

6. B. Capozzi, J. Xia, O. Adak, E. J. Dell, Z.-F. Liu, J. C. Taylor, J. B. Neaton, L. M. Campos, L. Venkataraman, Single-molecule diodes with high rectification ratios through environmental control. *Nat. Nanotechnol.* **10**, 522–527 (2015).

7. X. Chen, M. Roemer, L. Yuan, W. Du, D. Thompson, E. del Barco, C. A. Nijhuis, Molecular diodes with rectification ratios exceeding 10^5 driven by electrostatic interactions. *Nat. Nanotechnol.* **12**, 797–803 (2017).

8. L. Yuan, N. Nerngchamnong, L. Cao, H. Hamoudi, E. del Barco, M. Roemer, R. K. Sriramula, D. Thompson, C. A. Nijhuis, Controlling the direction of rectification in a molecular diode. *Nat. Commun.* **6**, 6324 (2015).

9. P. Reddy, S.-Y. Jang, R. A. Segalman, A. Majumdar, Thermoelectricity in molecular junctions. *Science* **315**, 1568–1571 (2007).

10. L. Cui, R. Miao, K. Wang, D. Thompson, L. A. Zotti, J. C. Cuevas, E. Meyhofer, P. Reddy, Peltier cooling in molecular junctions. *Nat. Nanotechnol.* **13**, 122–127 (2018).

11. S. Park, H. J. Yoon, New approach for large-area thermoelectric junctions with a liquid eutectic gallium-indium electrode. *Nano Lett.* **18**, 7715–7718 (2018).

12. H. Song, Y. Kim, Y. H. Jang, H. Jeong, M. A. Reed, T. Lee, Observation of molecular orbital gating. *Nature* **462**, 1039–1043 (2009).

13. L. Meng, N. Xin, C. Hu, H. A. Sabea, M. Zhang, H. Jiang, Y. Ji, C. Jia, Z. Yan, Q. Zhang, L. Gu, X. He, P. Selvanathan, L. Norel, S. Rigaut, H. Guo, S. Meng, X. Guo, Dual-gated single-molecule field-effect transistors beyond Moore's law. *Nat. Commun.* **13**, 1410 (2022).

14. Y. Li, W. Xu, Y.-L. Zou, J. Li, T. Gao, R. Huang, L. Chen, Z. Xiao, J. Shi, Y. Yang, W. Hong, Redox-mediated single-molecule transistor with a subthreshold swing down to 120 mV decade^{-1}. *Adv. Funct. Mater.* **33**, 2302985 (2023).

15. C. Tang, L. Huang, S. Sangtarash, M. Noori, H. Sadeghi, H. Xia, W. Hong, Reversible switching between destructive and constructive quantum interference using atomically precise chemical gating of single-molecule junctions. *J. Am. Chem. Soc.* **143**, 9385–9392 (2021).

16. C. Jia, M. Famili, M. Carlotti, Y. Liu, P. Wang, I. M. Grace, Z. Feng, Y. Wang, Z. Zhao, M. Ding, X. Xu, C. Wang, S.-J. Lee, Y. Huang, R. C. Chiechi, C. J. Lambert, X. Duan, Quantum interference mediated vertical molecular tunneling transistors. *Sci. Adv.* **4**, eaat8237 (2018).

17. X. Wang, A. Ismael, S. Ning, H. Althobaiti, A. Al-Jobory, J. Girovsky, H. P. A. G. Astier, L. J. O'Driscoll, M. R. Bryce, C. J. Lambert, C. J. B. Ford, Electrostatic Fermi level tuning in large-scale self-assembled monolayers of oligo(phenylene-ethynylene) derivatives. *Nanoscale Horiz.* **7**, 1201–1209 (2022).

18. M. Famili, C. Jia, X. Liu, P. Wang, I. M. Grace, J. Guo, Y. Liu, Z. Feng, Y. Wang, Z. Zhao, S. Decurtins, R. Häner, Y. Huang, S.-X. Liu, C. J. Lambert, X. Duan, Self-assembled molecular-electronic films controlled by room temperature quantum interference. *Chem* **5**, 474–484 (2019).

19. Y. Zhang, L. Liu, B. Tu, B. Cui, J. Guo, X. Zhao, J. Wang, Y. Yan, An artificial synapse based on molecular junctions. *Nat. Commun.* **14**, 247 (2023).

20. Y. Wang, Q. Zhang, H. P. A. G. Astier, C. Nickle, S. Soni, F. A. Alami, A. Borrini, Z. Zhang, C. Honnigfort, B. Braunschweig, A. Leoncini, D.-C. Qi, Y. Han, E. del Barco, D. Thompson, C. A. Nijhuis, Dynamic molecular switches with hysteretic negative differential conductance emulating synaptic behaviour. *Nat. Mater.* **21**, 1403–1411 (2022).

21. S. Goswami, R. Pramanick, A. Patra, S. P. Rath, M. Foltin, A. Ariando, D. Thompson, T. Venkatesan, S. Goswami, R. S. Williams, Decision trees within a molecular memristor. *Nature* **597**, 51–56 (2021).

22. J. Park, A. N. Pasupathy, J. I. Goldsmith, C. Chang, Y. Yaish, J. R. Petta, M. Rinkoski, J. P. Sethna, H. D. Abruña, P. L. McEuen, D. C. Ralph, Coulomb blockade and the Kondo effect in single-atom transistors. *Nature* **417**, 722–725 (2002).

23. W. Liang, M. P. Shores, M. Bockrath, J. R. Long, H. Park, Kondo resonance in a single-molecule transistor. *Nature* **417**, 725–729 (2002).

24. S. Tao, Q. Zhang, A. Vezzoli, C. Zhao, C. Zhao, S. J. Higgins, A. Smogunov, Y. J. Dappe, R. J. Nichols, L. Yang, Electrochemical gating for single-molecule electronics with hybrid Au|graphene contacts. *Phys. Chem. Chem. Phys.* **24**, 6836–6844 (2022).

25. X. Li, Y. Zheng, Y. Zhou, Z. Zhu, J. Wu, W. Ge, Y. Zhang, Y. Ye, L. Chen, J. Shi, J. Liu, J. Bai, Z. Liu, W. Hong, Supramolecular transistors with quantum interference effect. *J. Am. Chem. Soc.* **145**, 21679–21686 (2023).

26. N. Xin, X. Li, C. Jia, Y. Gong, M. Li, S. Wang, G. Zhang, J. Yang, X. Guo, Tuning charge transport in aromatic-ring single-molecule junctions via ionic-liquid gating. *Angew. Chem. Int. Ed.* **57**, 14026–14031 (2018).

27. A. Nitzan, Electron transmission through molecules and molecular interfaces. *Annu. Rev. Phys. Chem.* **52**, 681–750 (2001).

28. R. M. Metzger, Unimolecular electronics. *Chem. Rev.* **115**, 5056–5115 (2015).

29. G. D. Kong, H. Song, S. Yoon, H. Kang, R. Chang, H. J. Yoon, Interstitially mixed self-assembled monolayers enhance electrical stability of molecular junctions. *Nano Lett.* **21**, 3162–3169 (2021).

30. G. D. Kong, H. Song, S. Yoon, H. Kang, R. Chang, H. J. Yoon, Dynamic variation of rectification observed in supramolecular mixed mercaptoalkanoic acid. *Small* **20**, 2305997 (2024).

31. J. Jin, G. D. Kong, H. J. Yoon, Deconvolution of tunneling current in large-area junctions formed with mixed self-assembled monolayers. *J. Phys. Chem. Lett.* **9**, 4578–4583 (2018).

32. S. Chen, L. Li, C. L. Boozer, S. Jiang, Controlled chemical and structural properties of mixed self-assembled monolayers of alkanethiols on Au(111). *Langmuir* **16**, 9287–9293 (2000).

33. E. C. P. Smits, S. G. J. Mathijssen, P. A. van Hal, S. Setayesh, T. C. T. Geuns, K. A. H. A. Mutsaers, E. Cantatore, H. J. Wondergem, O. Werzer, R. Resel, M. Kemerink, S. Kirchmeyer, A. M. Muzafarov, S. A. Ponomarenko, B. de Boer, P. W. M. Blom, D. M. de Leeuw, Bottom-up organic integrated circuits. *Nature* **455**, 956–959 (2008).

34. M. Novak, A. Ebel, T. Meyer-Friedrichsen, A. Jedaa, B. F. Vieweg, G. Yang, K. Voitchovsky, F. Stellacci, E. Spiecker, A. Hirsch, M. Halik, Low-voltage p- and n-type organic self-assembled monolayer field effect transistors. *Nano Lett.* **11**, 156–159 (2011).

35. A. Ringk, X. Li, F. Gholamrezaie, E. C. P. Smits, A. Neuhold, A. Moser, C. Van der Marel, G. H. Gelinck, R. Resel, D. M. de Leeuw, P. Strohriegl, N-type self-assembled monolayer field-effect transistors and complementary inverters. *Adv. Funct. Mater.* **23**, 2016–2023 (2013).

36. H. Vančik, *Basic Organic Chemistry for the Life Sciences* (Springer, 2022).

37. S. Watcharinyanon, E. Moons, L. S. O. Johansson, Mixed self-assembled monolayers of ferrocene-terminated and unsubstituted alkanethiols on gold: Surface structure and work function. *J. Phys. Chem. C* **113**, 1972–1979 (2009).

38. T. Tian, P. Rice, E. J. G. Santos, C.-J. Shih, Multiscale analysis for field-effect penetration through two-dimensional materials. *Nano Lett.* **16**, 5044–5052 (2016).

39. C. A. Nijhuis, W. F. Reus, G. M. Whitesides, Mechanism of rectification in tunneling junctions based on molecules with asymmetric potential drops. *J. Am. Chem. Soc.* **132**, 18386–18401 (2010).

40. J. Lee, H. S. Kim, Y.-H. Kim, Multi-space excitation as an alternative to the Landauer picture for nonequilibrium quantum transport. *Adv. Sci.* **7**, 2001038 (2020).

41. J. Lee, H. Yeo, Y.-H. Kim, Quasi-Fermi level splitting in nanoscale junctions from ab initio. *Proc. Natl. Acad. Sci. U.S.A.* **117**, 10142–10148 (2020).

42. H. Son, J. Lee, T. H. Kim, S. Choi, H. Choi, Y.-H. Kim, S. Lee, Emergence of multiple negative differential transconductance from a WSe_2 double lateral homojunction platform. *Appl. Surf. Sci.* **581**, 152396 (2022).

43. T. H. Kim, J. Lee, R.-G. Lee, Y.-H. Kim, Gate- versus defect-induced voltage drop and negative differential resistance in vertical graphene heterostructures. *NPJ Comput. Mater.* **8**, 50 (2022).

44. L. Sun, K. Zhuo, Y. Chen, Q. Du, S. Zhang, J. Wang, Ionic liquid-based redox active electrolytes for supercapacitors. *Adv. Funct. Mater.* **32**, 2203611 (2022).

45. G. M. Ku, E. Lee, B. Kang, J. H. Lee, K. Cho, W. H. Lee, Relationship between the dipole moment of self-assembled monolayers incorporated in graphene transistors and device electrical stabilities. *RSC Adv.* **7**, 27100–27104 (2017).

46. W. N. Hansen, G. J. Hansen, Standard reference surfaces for work function measurements in air. *Surf. Sci.* **481**, 172–184 (2001).

47. J. M. Soler, E. Artacho, J. D. Gale, A. García, J. Junquera, P. Ordejón, D. Sánchez-Portal, The SIESTA method for ab initio order-*N* materials simulation. *J. Phys. Condens. Matter* **14**, 2745–2779 (2002).

48. J. P. Perdew, K. Burke, M. Ernzerhof, Generalized gradient approximation made simple. *Phys. Rev. Lett.* **77**, 3865–3868 (1996).

49. N. Troullier, J. L. Martins, Efficient pseudopotentials for plane-wave calculations. *Phys. Rev. B* **43**, 1993–2006 (1991).

50. M. Hegner, P. Wagner, G. Semenza, Ultralarge atomically flat template-stripped Au surfaces for scanning probe microscopy. *Surf. Sci.* **291**, 39–46 (1993).

51. L. Yuan, L. Jiang, C. A. Nijhuis, The drive force of electrical breakdown of large-area molecular tunnel junctions. *Adv. Funct. Mater.* **28**, 1801710 (2018).

52. G. Velpula, R. Phillipson, J. X. Lian, D. Cornil, P. Walke, K. Verguts, S. Brems, H. Uji-i, S. De Gendt, D. Beljonne, R. Lazzaroni, K. S. Mali, S. De Feyter, Graphene meets ionic liquids: Fermi level engineering via electrostatic forces. *ACS Nano* **13**, 3512–3521 (2019).
